# Calcitriol modifies tight junctions, improves barrier function, and reduces TNF‐α‐induced barrier leak in the human lung‐derived epithelial cell culture model, 16HBE 14o‐

**DOI:** 10.14814/phy2.15592

**Published:** 2023-04-11

**Authors:** Elizabeth Rybakovsky, Katherine M. DiGuilio, Mary Carmen Valenzano, Sophie Geagan, Kaithlyn Pham, Ronald N. Harty, James M. Mullin

**Affiliations:** ^1^ The Lankenau Institute for Medical Research Wynnewood Pennsylvania USA; ^2^ Department of Biology Drexel University Philadelphia Pennsylvania USA; ^3^ Department of Pathobiology and Microbiology University of Pennsylvania, School of Veterinary Medicine Philadelphia Pennsylvania USA

**Keywords:** calcitriol, claudin, ERK, lung, micronutrient, Sharpe‐Strumia Research Foundation, tight junction, tumor necrosis factor

## Abstract

Using the 16HBE 14o‐ human airway epithelial cell culture model, calcitriol (Vitamin D) was shown to improve barrier function by two independent metrics – increased transepithelial electrical resistance (TER) and reduced transepithelial diffusion of ^14^C‐D‐mannitol (*J*_m_). Both effects were concentration dependent and active out to 168 h post‐treatment. Barrier improvement associated with changes in the abundance of specific tight junctional (TJ) proteins in detergent‐soluble fractions, most notably decreased claudin‐2. TNF‐α‐induced compromise of barrier function could be attenuated by calcitriol with a concentration dependence similar to that observed for improvement of control barrier function. TNF‐α‐induced increases in claudin‐2 were partially reversed by calcitriol. The ERK 1,2 inhibitor, U0126, itself improved 16HBE barrier function indicating MAPK pathway regulation of 16HBE barrier function. Calcitriol's action was additive to the effect of U0126 in reducing TNF‐ α ‐induced barrier compromise, suggesting that calcitriol may be acting through a non‐ERK pathway in its blunting of TNF‐ α – induced barrier compromise. This was supported by calcitriol being without effect on pERK levels elevated by the action of TNF‐α. Lack of effect of TNF‐ α on the death marker, caspase‐3, and the inability of calcitriol to decrease the elevated LC3B II level caused by TNF‐α, suggest that calcitriol's barrier improvement does not involve a cell death pathway. Calcitriol's improvement of control barrier function was not additive to barrier improvement induced by retinoic acid (Vitamin A). Calcitriol improvement and protection of airway barrier function could in part explain Vitamin D's reported clinical efficacy in COVID‐19 and other airway diseases.

## Graphical abstract

Vitamin D utility in COVID‐19 infection may be due in part to improvement of airway epithelial barrier function and support of airway epithelial barrier function in "cytokine storm" situations. Remodeling of tight junctions is part of this process. Although the ERK‐1,2 pathway is involved in regulation of airway barrier function, Vitamin D is seemingly acting through a non‐ERK‐1,2 pathway to achieve its epithelial barrier support here.

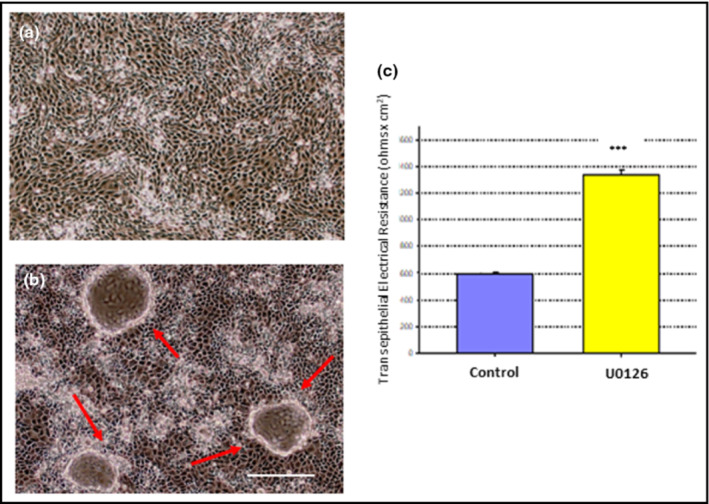

## 1 INTRODUCTION

There is now substantial published literature indicating that the current COVID‐19 epidemic is associated with an inverse correlation between Vitamin D status and COVID infection rates and severity. Patients exhibiting Vitamin D deficiency were 5‐fold more likely to manifest infection after adjusting for age (Katz et al., 2021). Deficiency also correlated with in‐patient mortality rates (Infante et al., 2021). The clinical data are less clear regarding Vitamin D *supplementation* (with frequent calls in the literature for expanded clinical trials [Murdaca et al., 2020]) but the highly favorable risk/benefit ratio for this micronutrient prompted recommendations for its immediate clinical use (Brenner & Schöttker, 2020). This current SARS‐CoV‐2‐driven interest in Vitamin D draws from a past interest in Vitamin D therapeutic and prophylactic utility for respiratory viruses in general. For a wide array of respiratory infections, serum levels of Vitamin D were observed to correlate with a decreased risk of infection (Sabetta et al., 2010). Notably, Vitamin D supplementation blunted inflammatory responses to influenza virus resulting in lower levels of TNF‐α, IL‐6, IL‐8, and IFN‐ɤ. These are all proinflammatory cytokines that would likely compromise airway barrier function and potentiate the clinical effects of a respiratory infection (Hayashi et al., 2020; Khare et al., 2013). To summarize, there is abundant and growing clinical interest in Vitamin D utility in respiratory infections‐ most pointedly with COVID‐19‐ that warrant further basic research into how Vitamin D might exert therapeutic and/or prophylactic benefit.

The importance of the role of a robust epithelial barrier in combating infectious diseases has been recognized in an ever‐increasing number of reviews on the subject (Groeger & Meyle, 2015; Guttman & Finlay, 2009; Mullin, Agostino, et al., 2005; Sawada, 2013; Torres‐Flores & Arias, 2015). Epithelial cell layers with their lynchpin tight junctional (TJ) complexes are obvious obstacles to infectious microorganisms. Less obvious are the mechanisms that these microorganisms have evolved to selectively target and damage the TJ complex and thereby compromise the barrier. From viruses to bacteria to dust mites, the TJ complex – and with it the barrier is perhaps the most common structural target in microbial infection. This is as true for the airway epithelium as it is for any other epithelial tissue (Inoue et al., 2020).

Modifying the TJ complex and improving barrier function have thus become potential prophylactic if not therapeutic options in airway infectious disease and diseases targeting epithelial barriers in general (Colpitts & Baumert, 2017; Krug et al., 2014; Valenzano et al., 2015).

In the past decade there has been an explosive increase in reports describing the ability of select micronutrients to structurally modify TJs and improve their barrier function (Bücker et al., 2020; Lee et al., 2019; Mohanty et al., 2020; Vargas‐Robles et al., 2019; Yamada & Kanda, 2019).

In addition, this has included the ability of select micronutrients to reduce the damaging effects of different aspects of the disease state on barrier function, such as the proinflammatory cytokine cascade (Krug et al., 2014). Vitamin D has been no exception here, with reports describing its ability to improve/protect barrier function in retinal, intestinal, and urinary bladder epithelial models (Fernandez‐Robredo et al., 2020; Lee et al., 2019; Mohanty et al., 2020). Specific effects of Vitamin D on airway barrier function have also been described (Chen et al., 2018; Li et al., 2015; Ma et al., 2020).

The 16HBE human cell culture provides at confluence a polar, differentiated, airway epithelial model with well‐described barrier properties, even though its subconfluent, cycling cells are poorly differentiated and non‐polar (Callaghan et al., 2020; Cozens et al., 1994; Haws et al., 1992; Uddin et al., 2008). Among their most notable differentiated characteristics are cell polarity with a microvillous apical cell surface (Zhu et al., 1999), apically situated tight junctions (Chowdhury et al., 2010), transepithelial voltage, short circuit current, substantial electrical resistance (Haws et al., 1992), and an apical CFTR chloride channel with transepithelial and unidirectional chloride secretion (Wine et al., 1994). Although 16HBE lacks true cilia expression, it does express the TRPV4 cation channel known to regulate cilia movement (Alenmyr et al., 2014). A wide range of investigators have extensively utilized 16HBE cell layers as models for airway function and in airway disease studies (Callaghan et al., 2020; Durgan et al., 2015; Sekiyama et al., 2012; Shintani et al., 2015; Sweerus et al., 2017; Xatzipsalti & Papadopoulos, 2007). This current study represents an investigation of Vitamin D improvement of barrier function of this airway epithelial barrier model, as well as its protection from a proinflammatory agent. It follows a recent report of similar activity by Vitamin A (retinoic acid; RA) in the same 16HBE model (Callaghan et al., 2020).

## 2 MATERIALS AND METHODS

### 2.1 Cell culture

The 16HBE 14o‐ (16HBE) cell culture was obtained from Millipore Sigma (St. Louis, MO) and used between passages 44–64 (Callaghan et al., 2020) before returning to frozen cell stocks. After reaching confluence, cells were trypsinized (0.25% trypsin, 2.21 mM EDTA) (Corning Cellgro) and then passaged on a weekly basis by seeding 1.5 × 10^6^ cells per Falcon 75 cm^2^ culture flask with 25 ml of Dulbecco's Modified Minimum Essential Medium, supplemented with 2 mM L‐Glutamine, 10% fetal bovine serum, 1% non‐essential amino acids, and 1 mM sodium pyruvate. Culture medium and additives were products of Corning Cellgro, except for the fetal bovine serum (Seradigm, VWR, Inc.). Cultures were incubated at 37°C in 95% air/5% CO_2_ humidified atmosphere.

### 2.2 Treatment with TNF‐α, calcitriol, retinoic acid and U0126

U0126 (Cell Signaling) was dissolved in DMSO to a 50 mM stock and then diluted directly into culture medium for the appropriate concentration. Matched control conditions used an equivalent amount of DMSO. Calcitriol (Enzo Life Sciences and Sigma Aldrich) and Retinoic Acid (Sigma Aldrich) were dissolved in ethanol at 50 uM and 33 mM respectively as stock solutions, to achieve final concentrations in culture medium of typically 50 nM and 50 uM (respectively).Matched control conditions for each micronutrient used an equivalent amount of ethanol. TNF‐α (Peprotech, Inc.) was prepared as a stock solution (100 ng/μl) in culture medium and then added to culture medium to a final concentration of 150 ng/ml. TNF‐α stocks were kept frozen at −80°C and thawed only once.

### 2.3 Transepithelial permeability measurements

Cells were seeded into sterile Millicell polycarbonate (PCF) cell culture inserts (30 mm diameter with 0.4 μm pore size [EMD Millipore]) on day 0 at a seeding density of 2.0 × 10^6^ cells/insert as described previously (Callaghan et al., 2020). Four Millicell PCF inserts were placed in 100 mm petri dishes. On days 1 and 3 post‐seeding, all cell layers were refed with control medium containing 50 U/ml penicillin and 50 μg/ml streptomycin (Corning Cellgro) (2 ml apical, 15 ml basal‐lateral). All treatments with calcitriol or TNF‐α were begun on day 6 (when the cell layer barrier was established). Cell layers were refed with fresh culture medium on the morning of experiments and allowed to incubate at 37°C for 90‐min prior to electrophysiological readings. Transepithelial potential difference was measured at 37°C using 1 M NaCl salt bridges in series with calomel electrodes. Transepithelial electrical resistance (TER) was measured at room temperature (RT) using 1 second, 40 μamp direct current pulses (through 1 M NaCl salt bridges in series with Ag/AgCl electrodes) in a custom‐ made Lexan chamber designed to hold the Millicell PCF inserts. Ohm's law was used to calculate TER (V = iR). Current‐passing and voltage‐measuring salt bridges were positioned above and below the center point of the cell layers.

Following TER measurements, the basal‐lateral medium was aspirated and replaced with 15 ml of medium containing 0.1 mM, 0.2 μCi/ml ^14^C‐D‐mannitol (Perkin‐Elmer) and incubated at 37°C. Triplicate 50 μl samples were taken from the basal‐lateral medium to determine the specific activity via liquid scintillation counting (LSC). Duplicate 250 μl samples were taken from the apical side at either 60 or 90 minutes for LSC to determine mannitol transepithelial flux rates (*J*_m_) (picomoles/min/cm^2^).

### 2.4 Immunoblot analyses

Cell layers were harvested from Millicell PCF inserts after washing five times in cold PBS. For examining, total cell lysates, 500 μl of ice‐cold lysis buffer with protease and phosphatase inhibitors were then added to each PCF (Callaghan et al., 2020). For analyzing TJ proteins in subcellular fractions, 600 μl of Buffer A with protease and phosphatase inhibitors (but without detergent) were added. The cell layer was physically scraped off the filter at 4°C. The resulting suspension was collected, flash‐frozen, and stored at −80°C. Once thawed, whole‐cell lysates were prepared by sonication and ultracentrifugation. Sonication was performed with the Fisher Scientific Sonic Dismembrator (Model 100), Setting 3. Ultracentrifugation was performed in a Beckman Model L‐80 with a Ti‐70 rotor at 1,09,000 *g* for 1 h at 4°C, for both whole cell lysates and particulate fractions. Samples of these lysates were analyzed by PAGE using a 10%–20% gradient Tris‐glycine gel (Invitrogen, a division of Thermo Fisher Scientific) at 120 V for 80 min. Cell layers harvested in Buffer A were treated similarly but after ultracentrifugation the supernatant (cytosolic fraction) was collected, followed by solubilization of the pellet (membrane/cytoskeletal fraction) in lysis buffer for separate PAGE analysis. Precision Plus Kaleidoscope Protein Standards (Bio‐Rad, Inc.) were included on each gel. Proteins were transferred at 30 V for 1 h from the gel to a nitrocellulose membrane. The membranes were then washed three times with PBS‐T (0.3% Tween‐20) for 10 min and blocked with 5% milk/PBS‐T at RT for 1 h. Membranes were incubated with the specific primary antibody (anti‐rabbit claudins −3 and −7, tricellulin, or occludin; anti‐mouse claudins‐1, −4 and −5 [Thermo Fisher Scientific]; anti‐rabbit claudin‐2 [Abcam]), at 0.5 μg/ml in 5% milk/PBS overnight at 4°C. (In each instance, a 1:1000 dilution of each primary antibody was used to probe the immunoblot and obtain the final images shown [each with different exposure times]). Membranes were again washed three times, 10 min each, with PBS‐T, and then incubated with the secondary antibody (rabbit anti‐mouse‐ or goat anti‐rabbit‐IgG antibody labeled with horseradish peroxidase [Southern Biotech]) for 1 hour at RT. Membranes were incubated with the specific primary antibody (anti‐rabbit claudins −3 and − 7, tricellulin, or occludin; anti‐mouse claudins‐1, −4 and −5 [Thermo Fisher Scientific]; anti‐rabbit claudin‐2 [Abcam]), at 0.5 μg/ml in 5% milk/PBS overnight at 4°C. The membranes were then again washed four times, 10 min each, with PBS‐T, and treated for 10‐ to 60‐s with Western Lightning Plus‐ECL chemiluminescence reagents (PerkinElmer). The membranes' protein band densities were quantified using the BioRad ChemiDoc Imaging System. The band densities of the experimentally treated cell samples were compared to averages of corresponding control cell samples. All data were expressed as the mean ± standard error of the mean and statistically analyzed using a paired Student's *t*‐test.

ERK and pERK were analyzed in whole cell lysates. Primary antisera to ERK were obtained from Cell Signaling, Inc. Antisera to pERK was a product of Thermo Fisher. Caspase‐3 and LC3B I/II were also analyzed in whole cell lysates. Caspase‐3 and LC3B I/II antisera were both purchased from Cell Signaling, Inc.

### 2.5 Phase contrast microscopy

For microscopy imaging, cells were seeded in 6 well (9.6 cm^2^) dishes at a density of 1 × 10^6^ cells per well. At confluence, cells were treated with U0126 for 24 h. Images (100×) were taken using a Nikon Diaphot inverted phase contrast microscope.

### 2.6 Statistics

Statistical significance in these studies was tested by means of two‐sided Student's *t* tests when comparing a single control group with a single experimental group, or one‐way ANOVA when multiple groups with sufficient sample sizes were being compared. In both cases, significance was claimed when *p* < 0.05.

## 3 RESULTS

**FIGURE 1:**
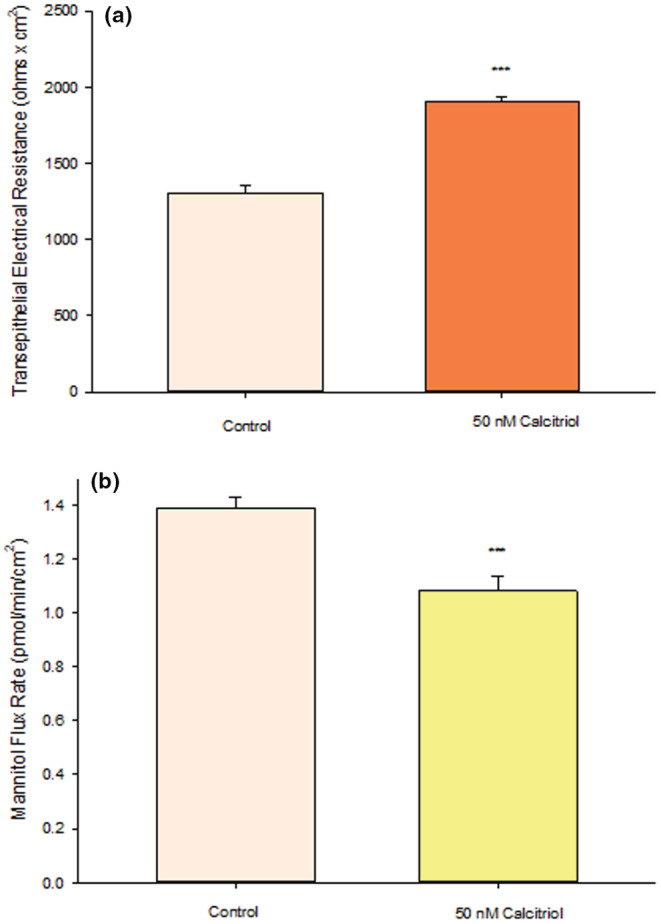
Effect of 50 nM Calcitriol on 16HBE Barrier Function. (a) TER and (b) transepithelial mannitol flux rate were completed as described in Material and Methods, 48 h after treatment. Data are represented as mean ± standard error for *n* = 12 cell layers per condition. ***p < 0.001. (Student's *t* test, two‐tailed).

**FIGURE 2:**
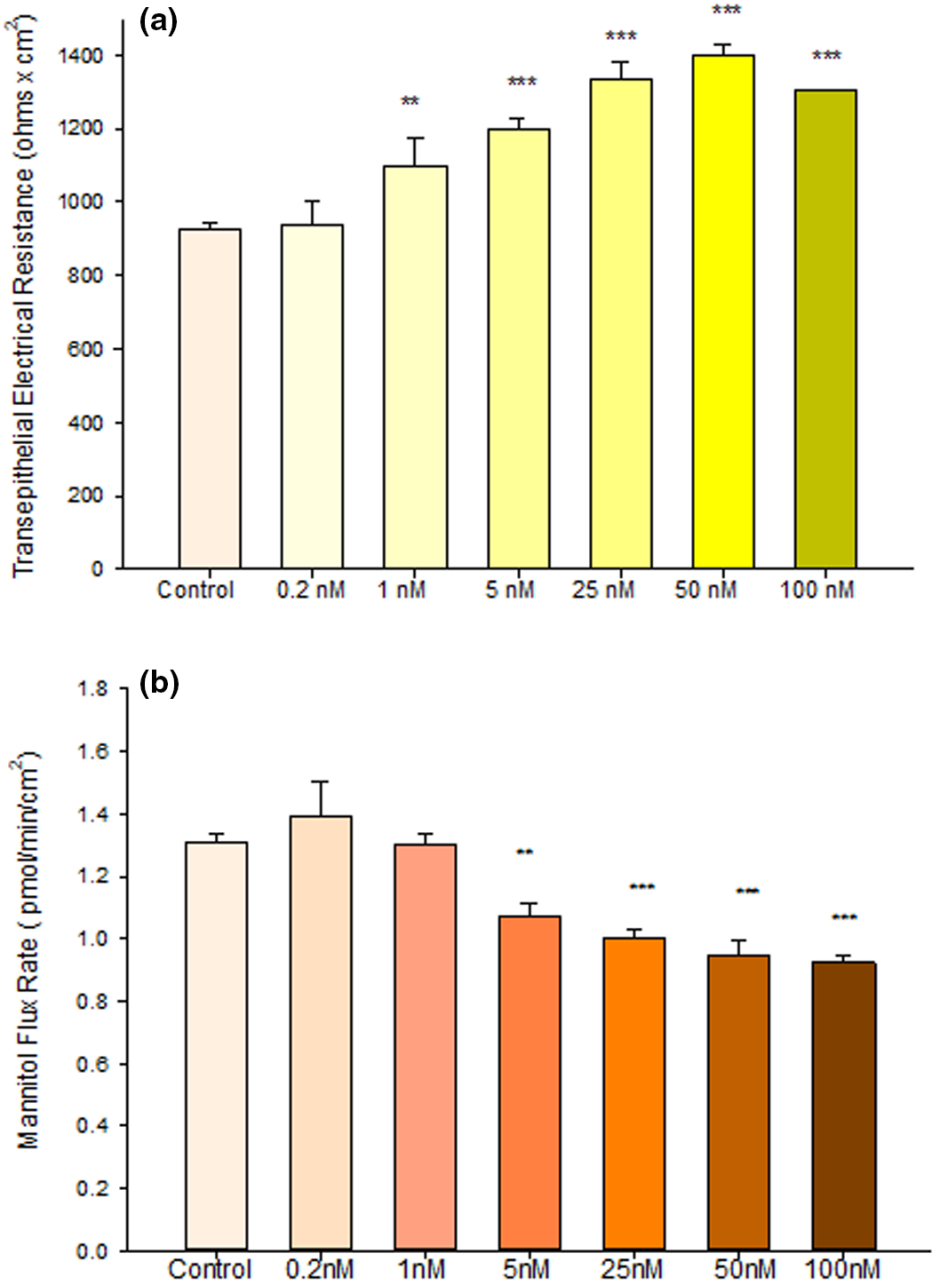
Concentration Dependence of Calcitriol Effects on 16HBE Barrier Function. (a) TER and (b) transepithelial mannitol flux rate were completed as described in Materials and Methods, 48 h after calcitriol treatment. *n* = 16 for control cell layers, *n* = 8 for 0.2 nM, 1 nM, 25 nM and 50 nM, *n* = 12 for 5 nM, *n* = 4 for 100 nM calcitriol ‐ treated cell layers. **p < 0.01, ***p < 0.001, One Way ANOVA, Holm‐Sidak Method.

**FIGURE 3:**
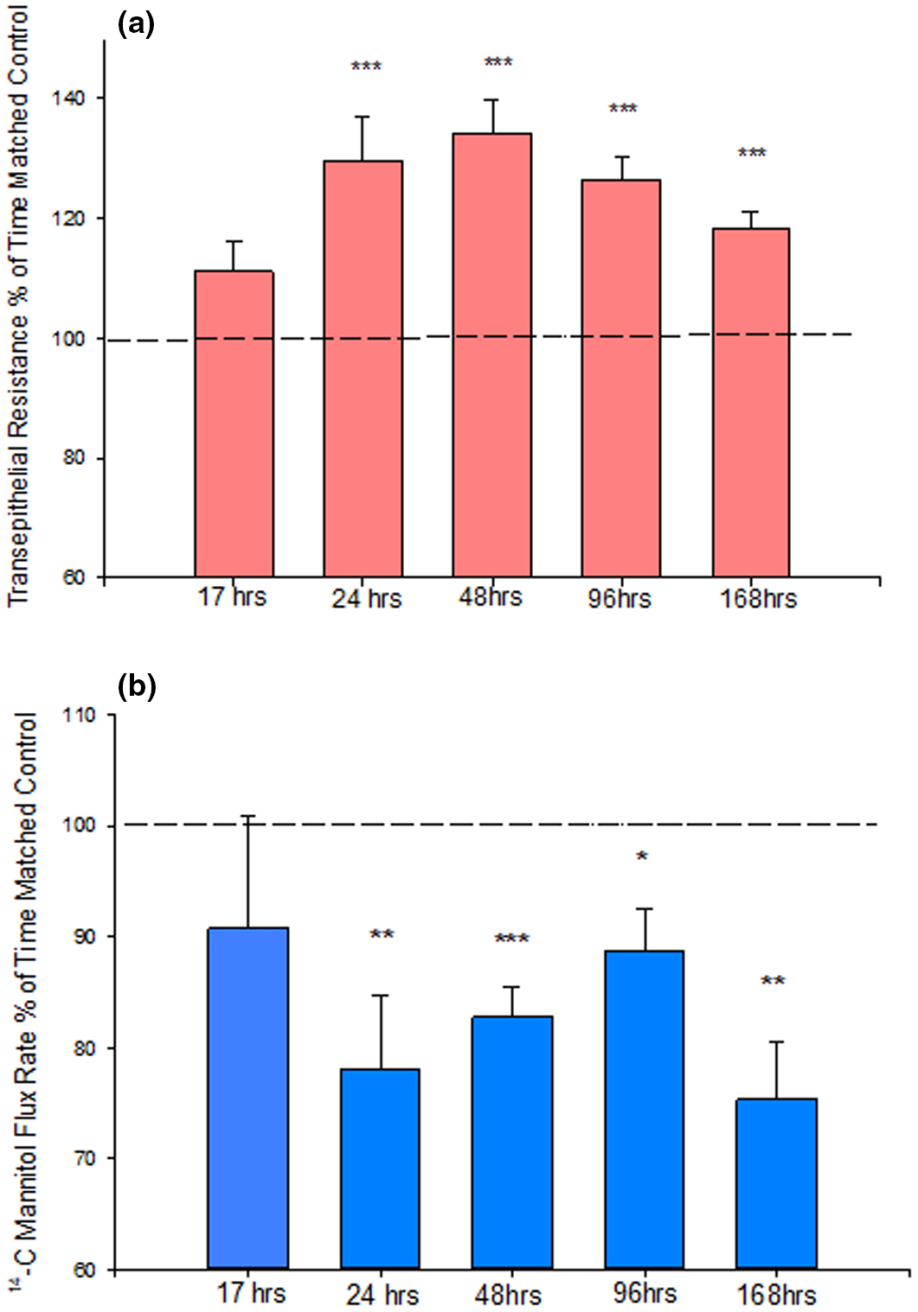
Time Course of Calcitriol Treatment on 16HBE Barrier Function. (a) TER and (b) transepithelial mannitol flux rate were completed as described in Materials and Methods at the appropriate time point following treatment with 50 nM calcitriol. *n* = 4 cell layers for 17, 168 h. *n* = 8 cell layers for 24, 48, 96 h. *p < 0.05, **p < 0.01, ***p < 0.001 versus time matched control. (Student's *t* test, two‐tailed). The dotted line indicates 100% (of time‐matched control), i.e. no effect.

**FIGURE 4:**
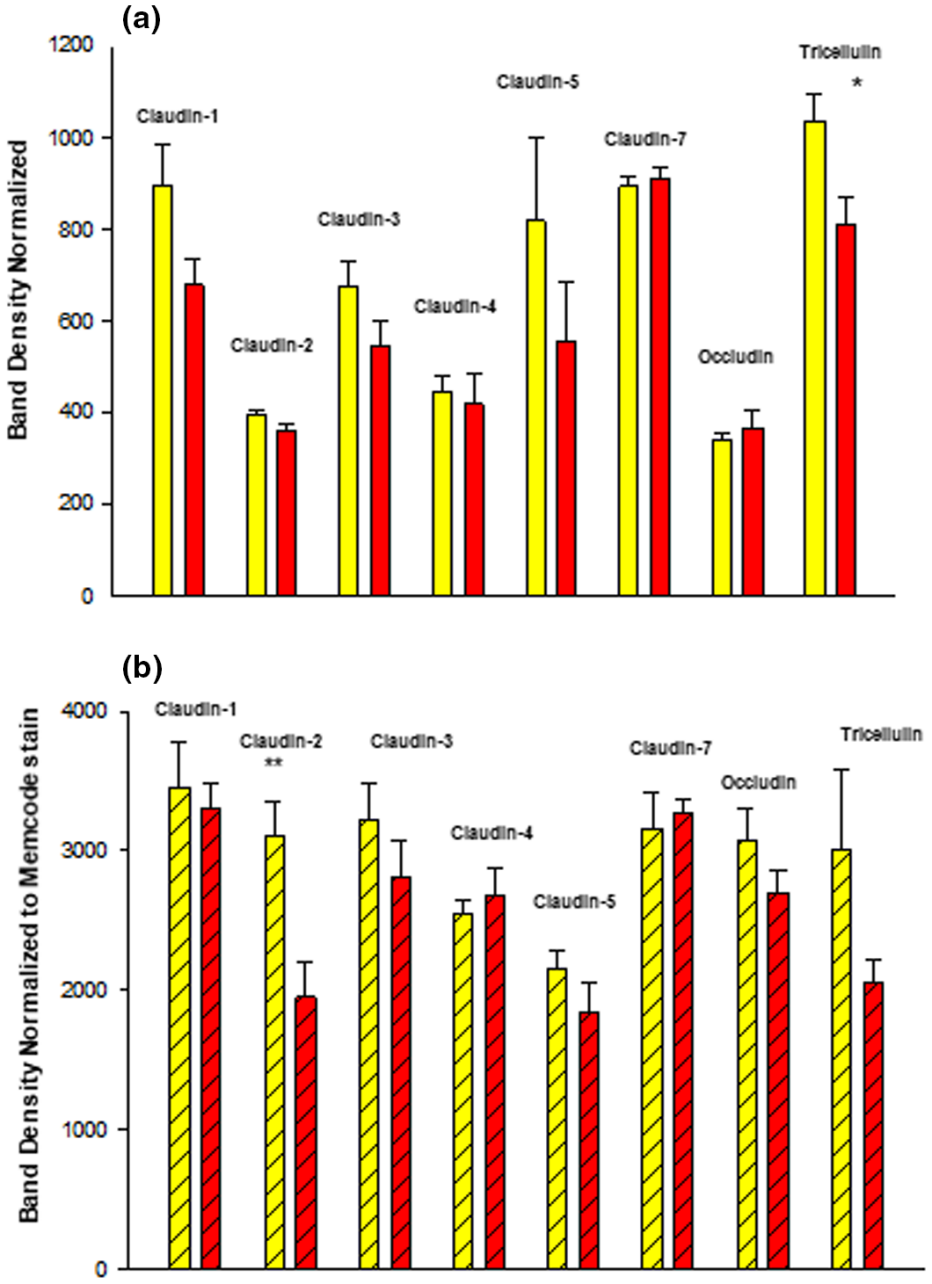
Effect of Calcitriol on 16HBE Tight Junctional Proteins. Confluent cell layers were treated for 48 h with 50 nM calcitriol, harvested, lysed and Western Immunoblots were performed for cytosolic (a) and detergent soluble fractions (b) as described in Material and Methods. Data are represented as mean ± standard error for *n* = 4 cell layers for each condition. **p < 0.01. (Student's *t* test, two‐tailed). Yellow bars indicate control cell layers, red bars indicate calcitriol ‐treated cell layers.

We observed that treatment with 50 nM calcitriol for 48 h significantly improved barrier function of 16HBE cell layers based upon two independent metrics. TER was increased significantly by 40% (Figure 1a) and transepithelial ^14^ C‐D‐Mannitol flux (*J*_m_) was decreased simultaneously by 25% (Figure 1b), both signifying decreased paracellular leak. As shown in Figure 2, a dose dependency of calcitriol was observed with maximal effects at a concentration of 50 nM. An increase in TER was observable at concentrations as low as 1 nM and peaked at 50 nM (Figure 2a). For ^14^C‐mannitol flux, we observed effects at calcitriol concentrations as low as 5 nM, with maximal decrease of leak also at 50 nM (Figure 2b). These barrier‐enhancing effects of 50 nM calcitriol were observed as early as 17 h post‐treatment and continued to increase until 48 h based upon TER data (Figure 3). However, the time‐courses of TER and mannitol flux differed. In Figure 3a, the TER increase was maximal at 48 h and then declined (though the increase was still significant at 168 h). However, a statistically significant decrease in *J*_m_ was as great at 168 h post‐treatment as it was at 24 h (Figure 3b). The statistically significant effects of 50 nM calcitriol on TER and *J*_m_ correlated with simultaneous changes in specific TJ proteins. This effect could be seen in Western immunoblots of junctional proteins in detergent‐soluble (particulate) subcellular fractions (Figure 4b) but not in corresponding cytosolic fractions (Figure 4a) from the same cell layer samples, where changes did not occur (with a possible exception of a decrease in tricellulin). Changes could likewise not be seen in analyses of TJ proteins in whole cell lysates. In the detergent‐soluble fractions, a statistically significant change (a 40% decrease) was seen only for claudin‐2. The TJ proteins, claudin‐1, −3, −4, −5, −7 and occludin did not exhibit any change in the detergent‐soluble fraction as a result of calcitriol treatment.

**FIGURE 5:**
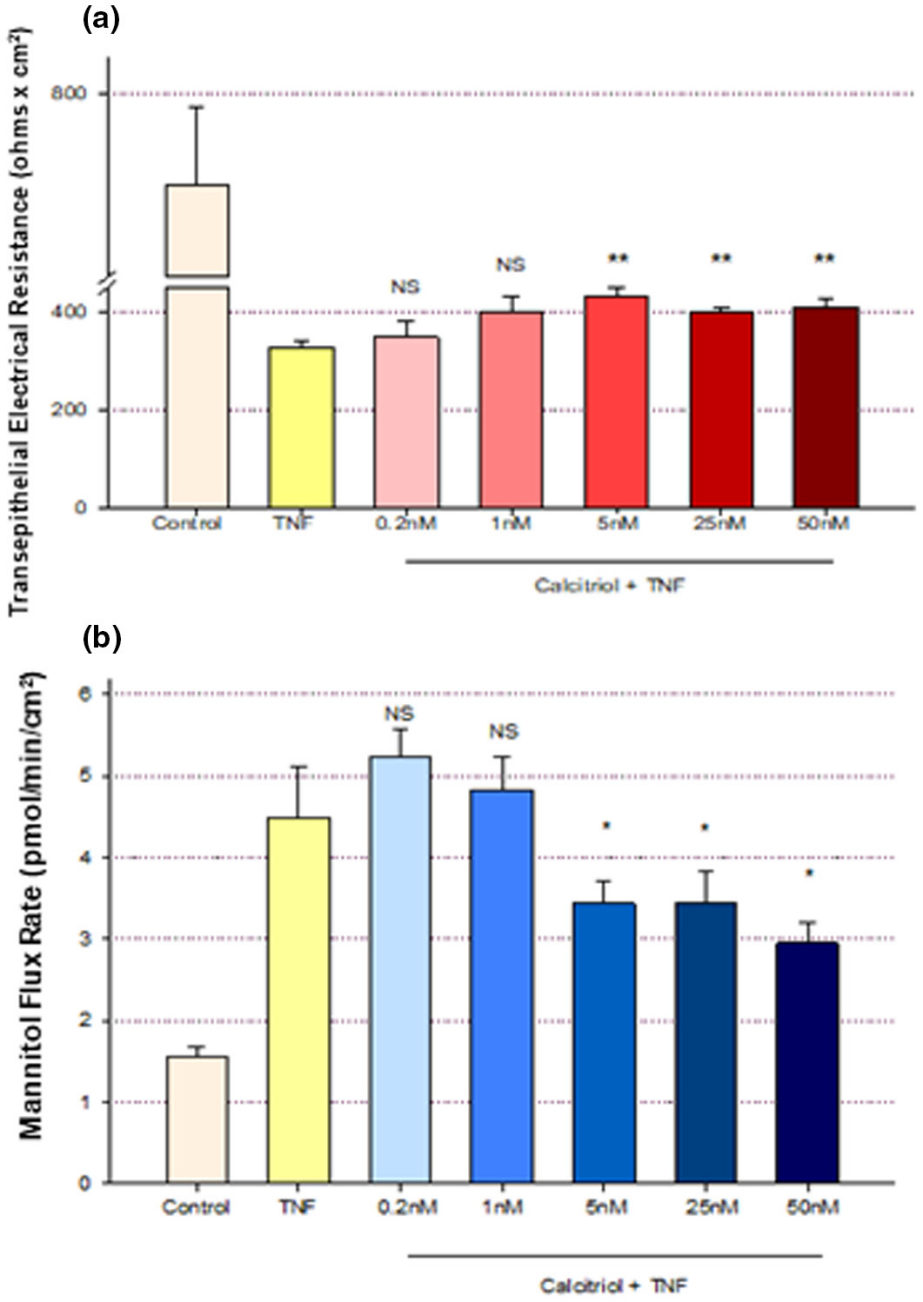
Effect of Calcitriol on TNF‐α‐Induced 16HBE Transepithelial Leak. (a) TER and (b) transepithelial mannitol flux rate were measured as described in Materials and Methods, 48 h post simultaneous treatment. Control and 150 ng/ml TNF‐α conditions had *n* = 6 cell layers, 50 nM calcitriol + TNF‐α had *n* = 14 cell layers. All other conditions had *n* = 8 cell layers. NS indicates no significant difference versus the TNF‐α condition. *p < 0.05, **p < 0.01 versus TNF‐α condition. (Student's *t* test, two‐tailed).

**FIGURE 6:**
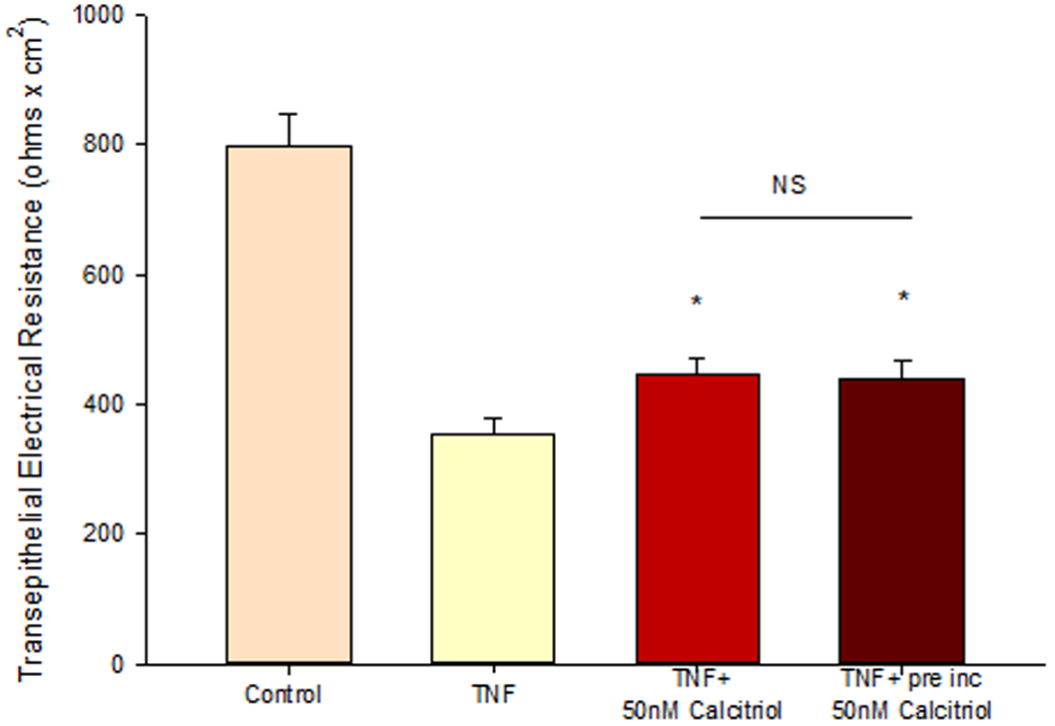
Effect of Preincubation with Calcitriol on TNF‐α‐Induced 16HBE Transepithelial Leak. TER was measured as described in Materials and Methods, 48 h post 150 ng/ml TNF‐α treatment. *n* = 4 cell layers per condition. *p < 0.05 versus TNF. NS indicates no significant difference between TNF‐α + calcitriol and TNF‐α + preincubation calcitriol. (Student's *t* test, two‐tailed.) “preinc” refers to 24 h preincubation with 50 nM calcitriol before the 48 h simultaneous TNF‐α + calcitriol treatment.

Callaghan et al. (2020) (Callaghan et al., 2020) showed that the proinflammatory cytokine, Tumor Necrosis Factor‐α (TNF‐α), decreases 16HBE barrier function as evidenced by reduced TER and increased *J*_m_. Retinoic acid was able to attenuate this induced leak by greater than 50%. In Figure 5, we observed that calcitriol also attenuated this TNF‐α‐induced leak. The inhibition of TNF‐α‐compromised barrier function seen with calcitriol was however weaker than what was observed with retinoic acid. The effects of TNF‐α on both TER and *J*_m_ were reduced approximately 20% and 30% respectively when treated simultaneously with TNF‐α and calcitriol for 48 h, compared to the changes seen with TNF‐α alone. (This is calculated based on the percent change from the TER and *J*_m_ value of TNF alone vs the change observed with TNF‐α and the maximal dose of calcitriol). Both measurements evidenced significant reduction of TNF‐α‐induced leak starting at a calcitriol concentration of 5 nM, similar to the concentration dependence of 16HBE barrier improvement by calcitriol in the absence of TNF‐ α (Figure 2). Moreover, 24 hr. preincubation with calcitriol did not induce any further protection from the barrier compromise seen with simultaneous treatment of calcitriol and TNF‐α (Figure 6). This experimental variation was performed to enable calcitriol to diffuse into the cell in advance of TNF‐α binding to its cell surface receptor.

**FIGURE 7:**
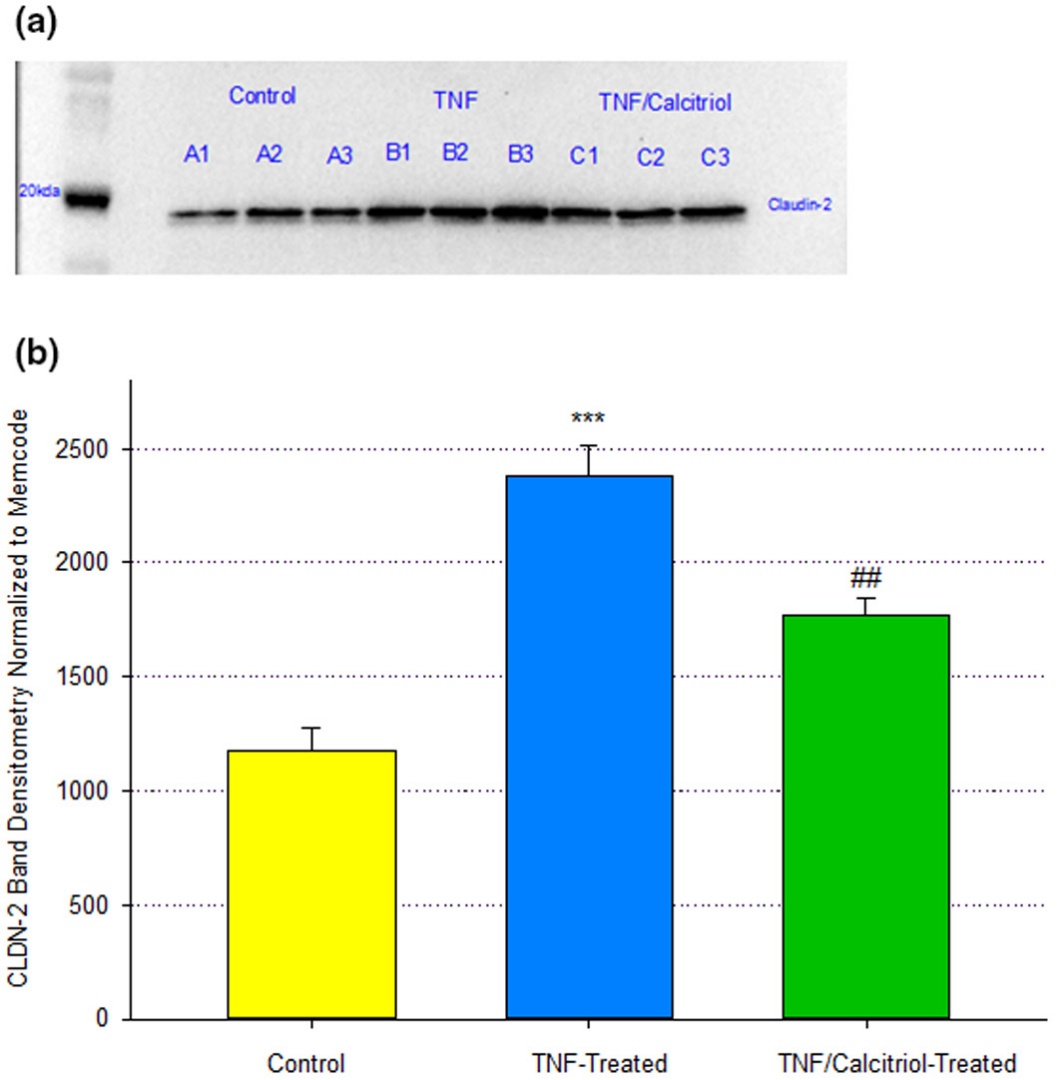
Effect of Calcitriol on TNF‐ α‐Induced Changes in 16HBE Claudin‐2 Levels. Confluent cell layers were treated with TNF‐α or calcitriol + TNF‐α for 48 h as described in Materials and Methods. (a) Control samples [lanes A1–A3], TNF‐ α‐treated samples [lanes B1–B3], TNF‐α + calcitriol‐treated samples [C1–C3]. (b) Optical densities of Claudin‐2 protein bands (*n* = 3 cell layers per condition). ***p < 0.001 versus control cell layers; ^##^P < 0.01 versus TNF‐α ‐treated cell layers. (Student's *t* test, two‐tailed).

Claudin‐2 offers an example of a TJ protein that changes in its abundance not only with respect to calcitriol treatment, but also with respect to calcitriol's effect on TNF‐ α ‐induced TJ changes in 16HBE cell layers (Figure 4b). It thereby indicates that calcitriol's ability to blunt the effect of TNF‐α on 16HBE barrier compromise is due at least in part to an effect on the TJ complex. Exposure of 16HBE cell layers to TNF‐ α for 48 h more than doubled claudin‐2 levels in particulate fractions of cell lysates (Figure 7). Simultaneous exposure of these cell layers to TNF‐α and 50 nM calcitriol significantly reduced this TNF‐α‐induced claudin‐2 elevation by almost 30%, although claudin‐2 levels remained elevated above control levels. This significant partial reversal of TNF‐ α effects on specific TJ proteins was however not observed with claudins −4, −5 or occludin.

**FIGURE 8:**
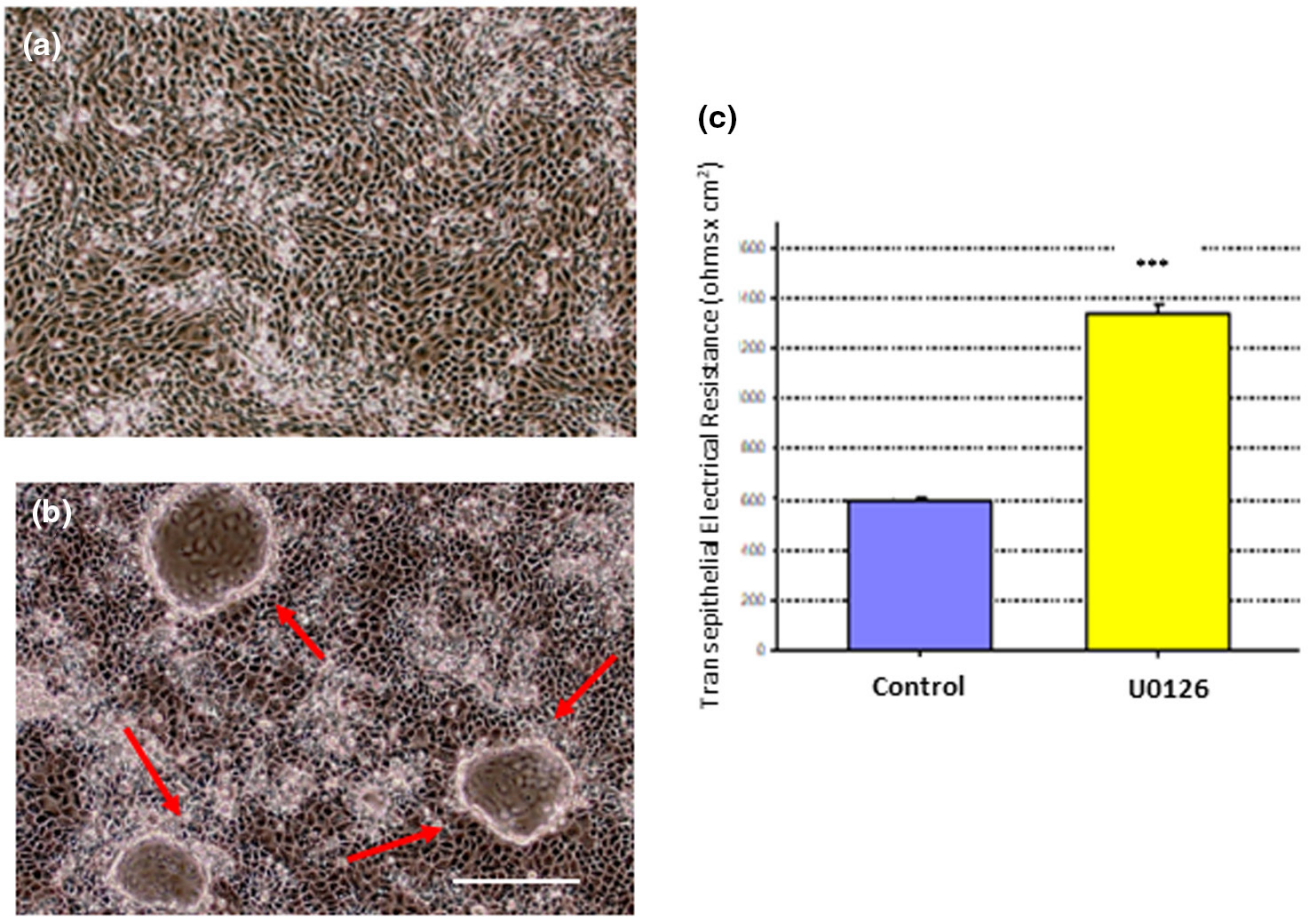
Effect of the ERK Inhibitor, U0126, on Dome Formation and Transepithelial Electrical Resistance in 16HBE cell layers. Phase contrast image of (a) control cell layers and (b) cell layers treated with 100 μM U0126 for 24 h. Red arrows indicate domes. Bar = 100 microns. TER (c) was measured 72 h post U0126 treatment as described in Material and Methods. *n* = 6 cell layers per condition. ***p < 0.001 versus control cell layers. (Student's *t* test, two‐tailed).

To investigate the potential role of the Raf/MEK/ERK pathway in regulation of 16HBE barrier function, we first used the well‐described ERK1/2 inhibitor, U0126. When 16HBE cell layers were treated with 100 μM U0126 for 48 h, a significant and dramatic increase in TER was observed (Figure 8c), evidence of ERK regulation of 16HBE barrier function. This improvement of TER was reflected in a change in confluent cell layer morphology shown in Figure 8a,b. 24 hr treatment with U0126 dramatically increased dome formation in the confluent 16HBE monolayer, consistent with the observed increase of 16HBE TER by U0126.

**FIGURE 9:**
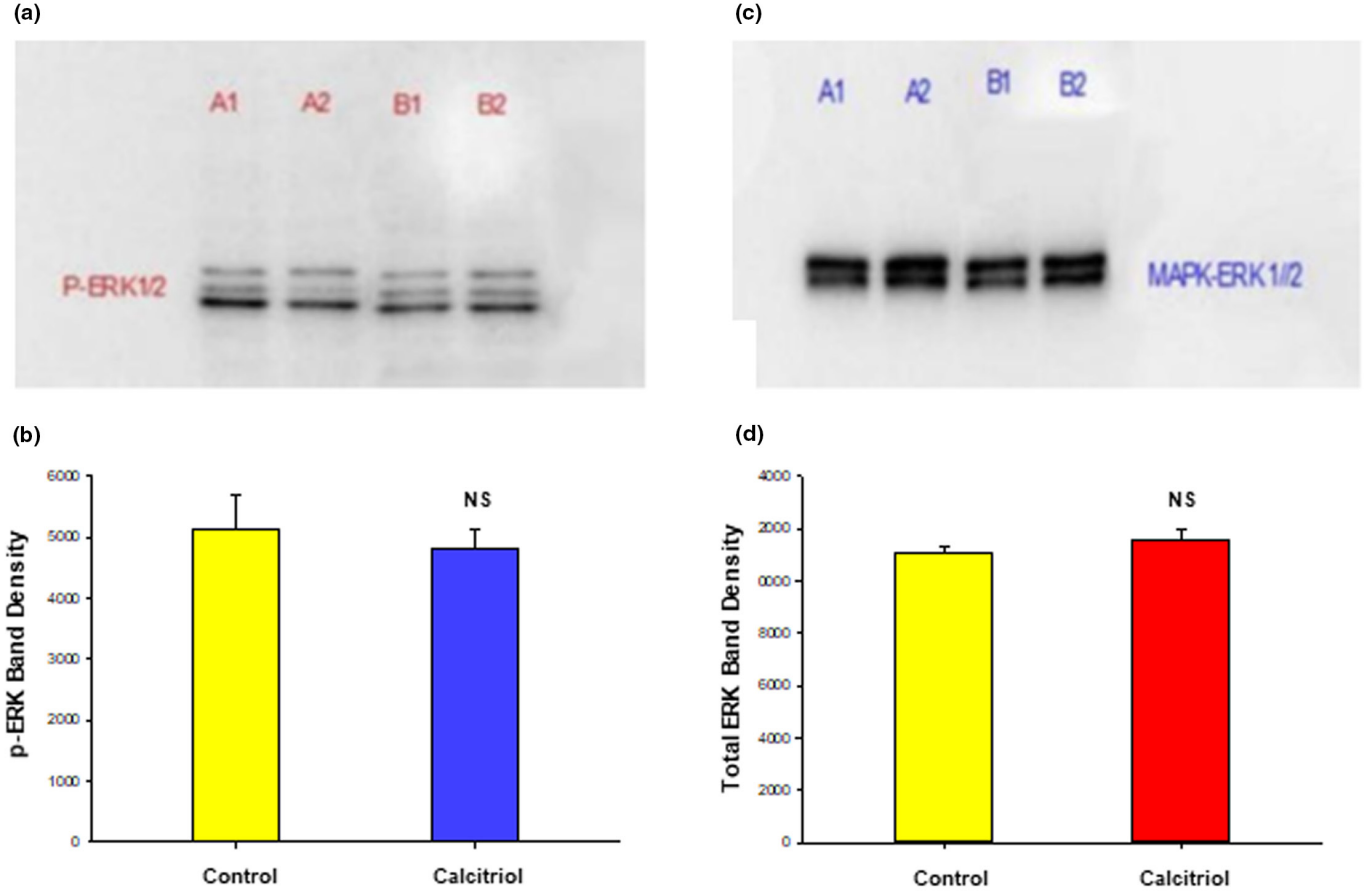
Lack of Effect of Calcitriol on 16HBE pERK Levels. Confluent cell layers were treated with control or 50 nM calcitriol‐supplemented media for 24 h prior to an additional treatment with respective media for 30 min as described in Materials and Methods. Cell layers were harvested, lysed and PAGE immunoblots were performed for phosphorylated‐ERK‐1/2 (a) and total ERK‐1/2 (c). Lane A: control; lane B: calcitriol‐treated. Two of three sample lanes are shown. Band densities of 3 separate cell samples were quantified as described in Materials and Methods (b and d). Bars represent mean ± SEM for 3 cell layers. NS = not significant vs control cell layers. (Student's *t* test, two‐tailed).

However, treatment of 16HBE cell layers with calcitriol, at a concentration and timeframe that gave consistent physiological effects, did not show any significant effect on pERK levels in these same cell layers, suggesting that the calcitriol effect on barrier function is not proceeding through the Raf/MEK/ERK pathway. As shown in Figure 9a,b, calcitriol did not affect the level of pERK in Western immunoblots of whole cell lysates of cell layers exhibiting calcitriol‐induced barrier improvement. There was also no effect on total ERK (Figure 9c,d).

**FIGURE 10:**
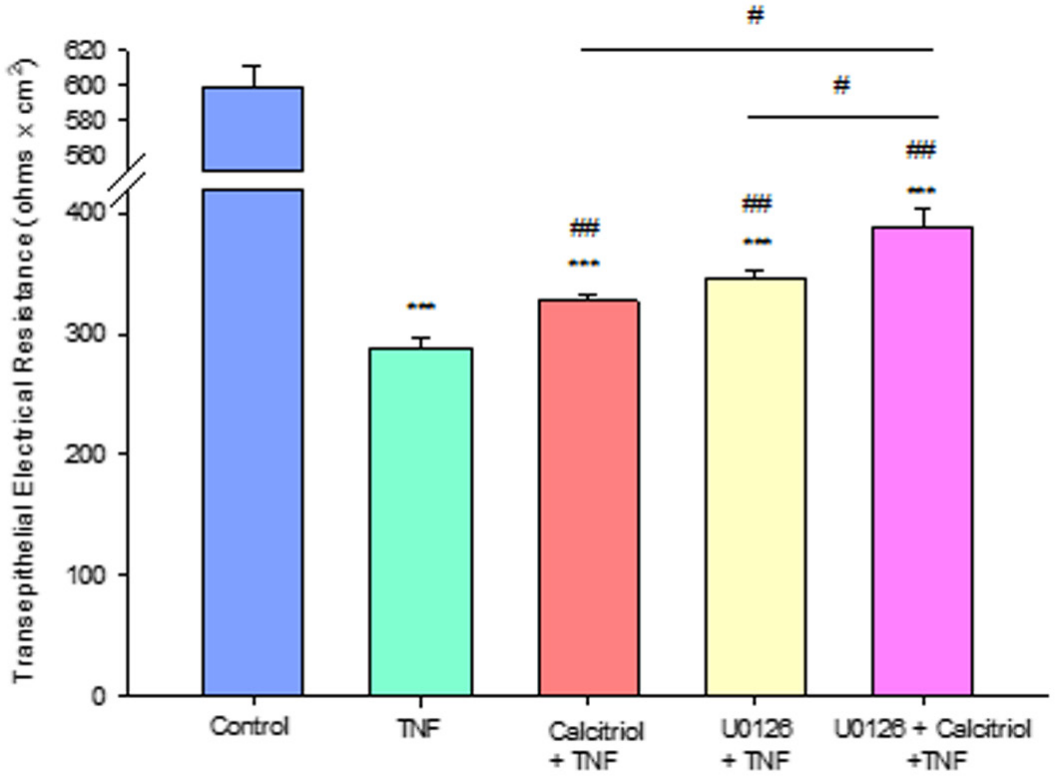
U0126 and Calcitriol Have Additive Effects on TNF‐α‐Induced Compromise of Transepithelial Electrical Resistance Across 16HBE Cell Layers. Cell layers were pretreated for 24 h. with 100 μM U0126 and/or 50 nM calcitriol followed by a 48‐hour treatment with 150 ng/ml TNF‐α ± U0126 and/or calcitriol. TER was then measured as described in Material and Methods. *n* = 6 cell layers per condition ± SEM. ***p < 0.001 versus control, ^##^ *p* < 0.01 versus TNF‐α, ^#^ *p* < 0.05 versus Vit D + TNF‐α or U0126 + TNF‐α. (Student's *t* test, two‐tailed).

In the presence of TNF‐ α, calcitriol may be acting through a non‐ERK pathway. As shown in Figure 10, the dramatic, greater than 50% decrease in 16HBE cell layer TER caused by TNF‐ α exposure, was partially reversed by U0126 as well as by calcitriol. Simultaneous treatment of TNF‐α‐treated cell layers with both U0126 and calcitriol produced an additive increase, significantly above the increase ensuing from U0126 or calcitriol alone. Taken together, this suggests that a non‐ERK pathway may be involved in the protective action of calcitriol on TNF‐ α ‐treated cell layers.

**FIGURE 11:**
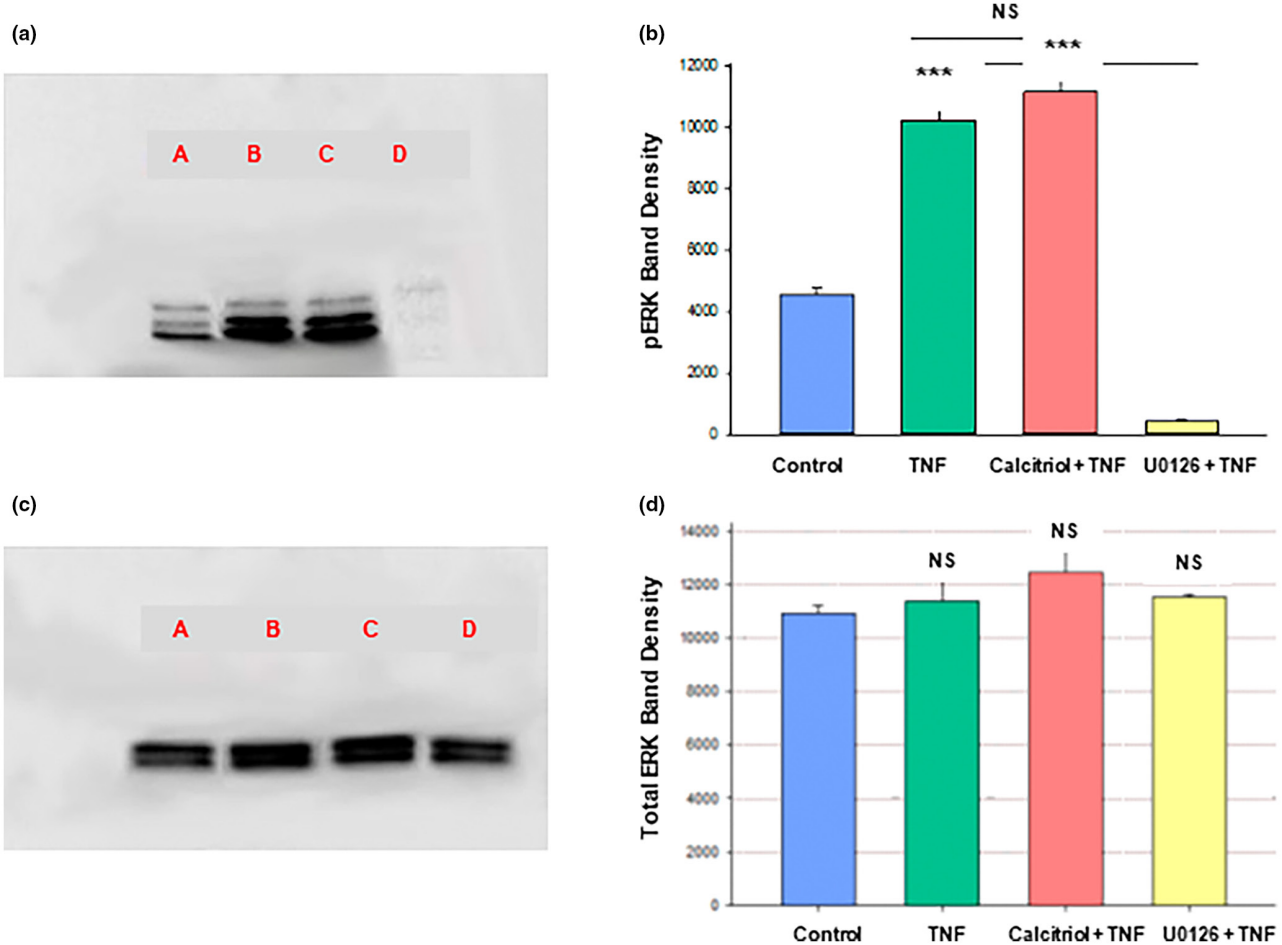
Effect of Calcitriol and U0126 on the TNF‐α‐Induced Increase of 16HBE pERK Levels. Confluent cell layers were treated with control or 50 nM calcitriol‐supplemented media for 24 h prior to treatment with control, 150 ng/ml TNF‐α alone, TNF‐α + 50 nM calcitriol or TNF‐α + 100 μM U0126 for 30 minutes as described in Materials and Methods. Cell layers were harvested, lysed and PAGE/immunoblots were performed for phosphorylated‐ERK‐1/2 (a) and total ERK‐1/2 (c). Lane A: control; lane B: TNF‐α ‐treated; lane C: calcitriol + TNF‐α –treated; lane D: U0126 + TNF‐α ‐treated. One of three sample lanes are shown. Band densities for 3 separate immunoblots were quantified as described in Materials and Methods (b and d). Bars represent mean ± SEM for 3 cell layers. ***p < 0.001 versus control; NS (not significant) versus TNF‐α (b) or versus control (d), ^###^ *P* < 0.001 versus TNF‐α (b). (Student's *t* test, two‐tailed).

Figure 11a,b provide further support for calcitriol working through a non‐ERK pathway to reduce the barrier compromise caused by TNF‐α, because here it can be seen that the pronounced (greater than 100%) increase in pERK induced by TNF‐ α is unaffected by calcitriol. U0126 on the other hand dramatically reduced pERK levels. Neither TNF‐ α, calcitriol nor U0126 were seen to affect levels of total ERK (Figure 11c,d).

**FIGURE 12:**
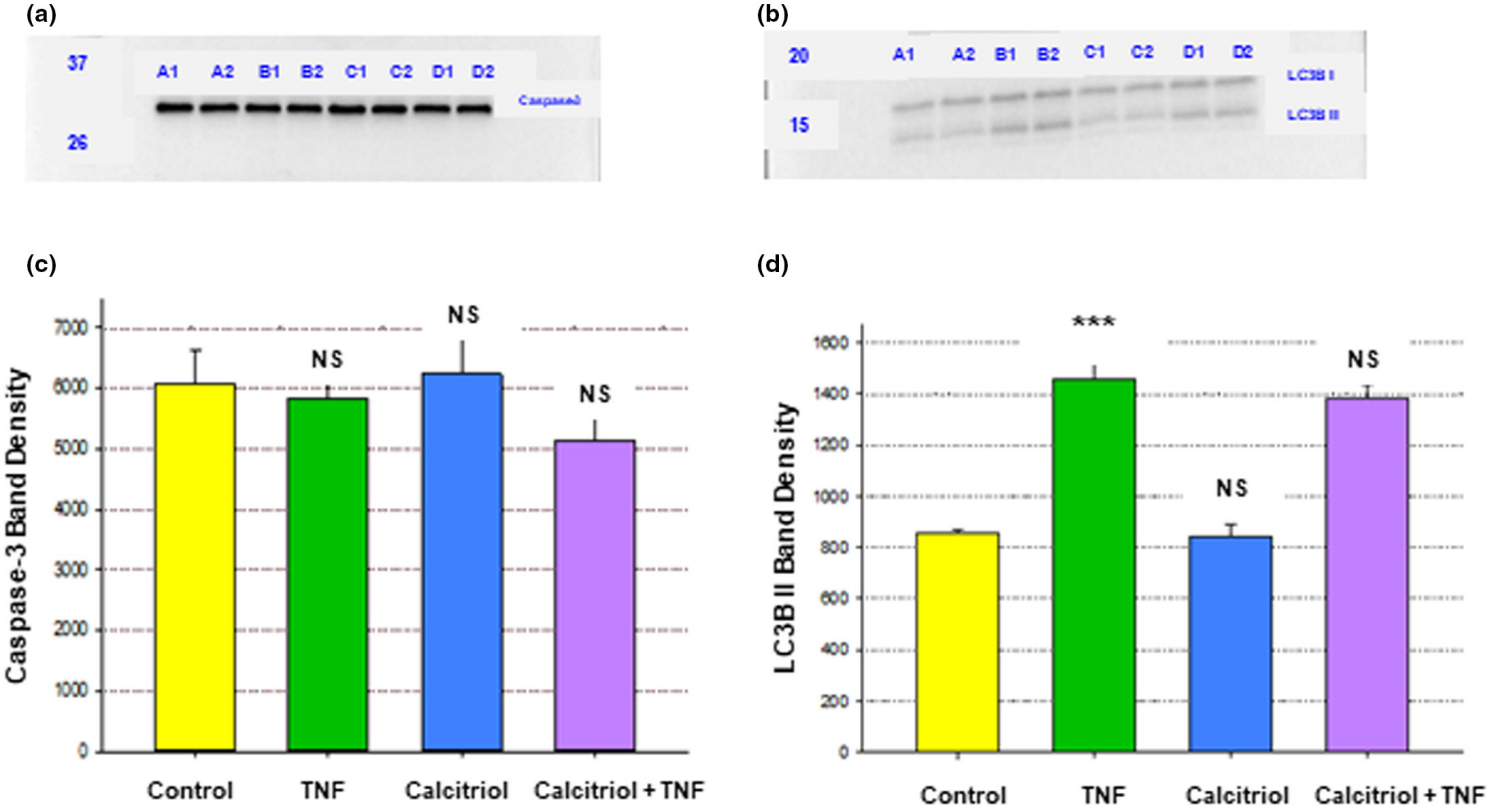
Effect of TNF‐ α and Calcitriol on 16HBE Caspase‐3 and LC3B Levels. Confluent cell layers were treated with calcitriol, TNF‐α, or calcitriol + TNF‐α for 48 h as described in Materials and Methods. (a) Caspase‐3 and (b) LC3B‐I and ‐II (Control samples [lanes A1–A2], TNF‐ α‐treated samples [lanes B1–B2], calcitriol‐treated samples [lanes C1–C2], TNF‐α + calcitriol‐treated samples [lanes D1–D2]). Optical densities of caspase‐3 (c) and LC3B‐II (d) protein bands (*n* = 3 cell layers per condition (the enclosed blots [a & b] showing 2 of 3 cell layers); ***p < 0.001 versus control cell layers; NS = not significant versus control cell layers or no significant difference between the TNF‐α and calcitriol + TNF‐α conditions. (Student's *t* test, two‐tailed).

Since the calcitriol modification of TNF‐α ‐induced barrier compromise apparently did not proceed via the ERK pathway, the possibility existed for calcitriol affecting barrier function by TNF‐α ‐induced cell death within the epithelial barrier. Cell death is a highly obvious source of epithelial barrier compromise (although the exact context in which it occurs can determine whether it actually contributes to barrier compromise) (Gitter et al., 2000; Peralta Soler et al., 1996). We therefore tested for calcitriol effects on 16HBE cell death by testing for calcitriol effects on the apoptotic marker, caspase‐3, and the autophagy markers, LC3B‐I/II. As shown in Figure 12a,c, however, TNF‐α had no significant effect on 16HBE caspase‐3 levels, suggesting a lack of TNF‐α ‐induced apoptosis for 16HBE cell layers, and thus seemingly ruling out apoptosis as a means by which calcitriol could be modifying the TNF‐α ‐induced decrease of barrier function. However, TNF‐α induced a significant increase in 16HBE LC3B‐II levels at 48 h, suggesting that TNF‐α ‐induced autophagy could be contributory to barrier compromise here. But calcitriol did not reduce the LC3B‐II elevation caused by TNF‐α (Figure 12b,d), suggesting that calcitriol's reduction of TNF‐α ‐induced barrier compromise was not proceeding via effects on autophagic cell death.

**FIGURE 13:**
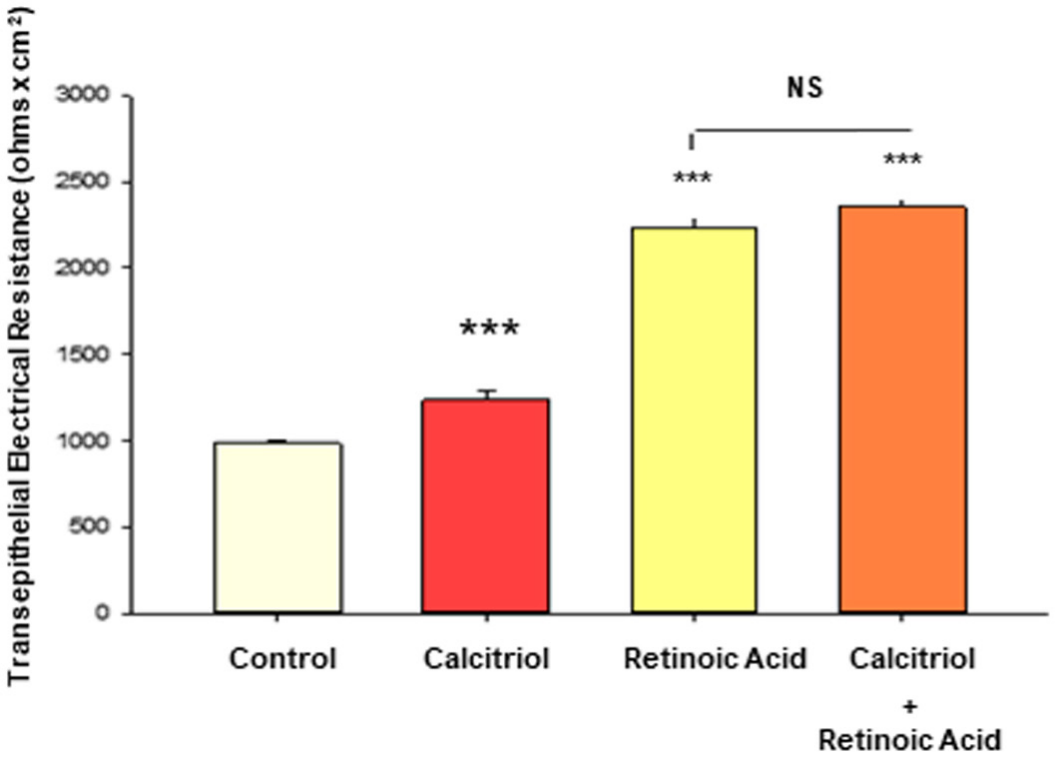
Effect of Retinoic Acid and Calcitriol on 16HBE Barrier Function. TER was performed as described in Material and Methods, 48 h after treatment with 50 nM calcitriol and/or 50 μM retinoic acid. *n* = 20 for control and retinoic acid cell layers, *n* = 28 for calcitriol cell layers, *n* = 32 for calcitriol + retinoic acid cell layers. ***p < 0.001 versus control condition. NS indicates no significant difference for retinoic acid vs combination conditions (Student's *t*‐test, two‐tailed).

It has been reported that retinoic acid and calcitriol can have additive effects on certain cell and tissue properties (Anand & Kaul, 2003; Cantorna et al., 2019; Surman et al., 2016). We had earlier demonstrated dramatic improvement of 16HBE barrier function by retinoic acid (Callaghan et al., 2020). In Figure 13, however, no additive effect was seen on TER after simultaneous treatment with 50 μM retinoic acid and 50 nM calcitriol, the concentrations at which both micronutrients exert maximal effects on 16HBE barrier function.

## 4 DISCUSSION

Our group recently reported the improvement of epithelial barrier function by retinoic acid (RA), as well as RA's ability to reduce TNF‐ α‐ induced barrier leak in the 16HBE 14o‐ airway epithelial model (Callaghan et al., 2020). We expand on that work in our current investigation, now showing a calcitriol‐induced improvement of barrier function in this same human airway epithelial cell culture model. Calcitriol significantly increased 16HBE barrier function as evidenced by a 40% increase in TER and a 25% decrease in transepithelial leak of ^14^C‐D‐mannitol (Figure 1). This was a concentration dependent decrease in paracellular leak (Figure 2) that could be observed by 17 h and continued through 7 days (Figure 3). This is in accordance with previously mentioned literature showing that Vitamin D is capable of both improving and protecting barrier function in several epithelial models including retinal, intestinal, and urinary bladder (Chen et al., 2018; Fernandez‐Robredo et al., 2020; Lee et al., 2019; Li et al., 2015; Ma et al., 2020; Mohanty et al., 2020).

It should be mentioned that the DMEM medium used to grow and maintain the 16HBE epithelial has no Vitamin D or Vitamin A content of its own. The only Vitamin A and Vitamin D in our final culture medium would come from the Fetal Bovine Serum (FBS) component of the culture medium. However, not only are these levels far below what we supplement in our studies, but our use of 10% FBS reduces these contributions even further. FBS has been reported to contain 500 nM Vitamin A and approximately 40 nM Vitamin D (Nelson et al., 2016; Randolph & Simon, 1993). Using 10% FBS in our 16HBE culture medium would result in a Vitamin A level of 50 nM and a Vitamin D level of 4 nM. These levels are far below our reported supplemented levels of 50 μM and 50 nM for Vitamins A and D respectively. In fact, the concentration dependence of Figure 2 for calcitriol could not have exemplified the nicely graduating effects of increasing concentration if we had started with a high baseline level of Vitamin D in the culture medium. The fact that barrier‐improving activity of key micronutrients occurs at levels above those normally present in serum (and in Recommended Daily Allowances) is a topic discussed in a recent review from our group (DiGuilio et al., 2022).

Correlating with the significant effects of calcitriol on barrier function, a change in the amount of one specific TJ protein, claudin‐2, could be observed in detergent‐soluble fractions of the cells after calcitriol exposure, although occludin and claudins −1, −3, −4, −5 and −7 were unaffected (Figure 4). Cytosolic fraction levels of all of these claudins were unaffected by calcitriol, and what is noteworthy in a procedural sense is that the change in claudin‐2 could not be observed in whole cell lysates (data not shown). Overall, these data indicate that changes in barrier function induced by calcitriol can be attributed at least in part to induced changes in TJ complexes. This situation is similar to our previous study with RA, where RA treatment dramatically increased 16HBE barrier function and also modified its TJ proteins, notably increasing levels of claudin‐4 by over 60% in Western immunoblots of cell layer lysates (Callaghan et al., 2020). Worth noting here is that Li et al. (2015) (Li et al., 2015) reported a lack of effect of Vitamin D treatment of 16HBE cell layers on levels of the tight junctional proteins, ZO‐1 and occludin in whole cell lysates.

Calcitriol's partial inhibition of the barrier compromise of 16HBE cell layers by TNF‐α (Figure 5) also correlated with changes in the level of abundance of at least one TJ protein. As shown in Figure 7, TNF‐α‐treatment (48 h) significantly increased the level of claudin‐2 in the detergent‐soluble fraction. Simultaneous treatment with TNF‐α and calcitriol resulted in a significantly lower level of claudin‐2, below the level achieved with TNF‐α treatment alone. This provides further substantiation that calcitriol is achieving its barrier function effects at least in part through modification of the 16HBE TJ complexes.

The initial discovery and description of claudins showed that these proteins localized to the region of tight junctions and actually constituted tight junction strands (Furuse et al., 1999). Later studies would show that claudins were conferring true barrier function on a cell layer in terms of transepithelial electrical resistance and inhibition of transepithelial paracellular diffusion (Sonoda et al., 1999). Many years of research from many laboratories would generate a concept regarding claudins as either pore‐forming (increasing barrier permeability) or sealing (decreasing barrier permeability), with claudins −2 and −10 being premier “pore formers” and claudins −1, −3 and −4 being premier “sealing claudins” (Rosenthal et al., 2017). However, it was also known that claudins have the ability to self‐associate not only in homomeric pairings but also in heteromeric pairings as well (Furuse et al., 1999). That ability of the 20+ members of the claudin family to engage in heteromeric interactions suggests that labeling specific claudins in one class or the other may however be somewhat complicated as it suggests that each claudin's functionality is dependent upon molecular associations with neighboring – and potentially different ‐ claudins. Consider that 20+ different claudins engaging in various heteromeric pairing creates a great deal of permutations. Those heteromeric interactions are highlighted by recent studies showing a functional interaction between claudins −5 and −18 as well as claudins −2 and −4 (Schlingmann et al., 2016; Shashikanth et al., 2022). That being said, our results however do support the canonical view in that the increased TER observed for calcitriol treatment of 16HBE cell layers did correspond in our study with significantly decreased claudin‐2 levels in particulate fractions (Figure 4b), what one would predict from a “pore‐forming” claudin. Regarding TNF‐α‐induced decrease of TER, we observed a significant increase in claudin‐2 levels with TNF‐α exposure (Figure 7), an increase that was significantly reduced by simultaneous exposure of TNF‐α and calcitriol. Again, claudin‐2 was behaving here as a paradigm pore‐forming claudin since its increase with TNF‐α correlates with a sharp decrease in TER, while co‐incubation with TNF‐α and calcitriol significantly increased TER above the level seen with TNF‐α alone. TNF‐α is well known to increase claudin‐2 while decreasing barrier function (Droessler et al., 2022). The kinetics of the effects on claudin‐2 can however be complex (Amoozadeh et al., 2015). Vitamin D has been observed to correlate with reduced expression of claudin‐2 (Meckel et al., 2016; Pan et al., 2012).

Li et al. (2015) (Li et al., 2015) had earlier shown that 1,25‐dihydroxyvitamin D3 attenuated the effect of toluene diisocyanate‐induced airway barrier disruption through the ERK pathway. ERK activity has been shown to negatively regulate epithelial barrier integrity in a variety of epithelial models including lung (Aggarwal et al., 2011; Barbin et al., 2001; Mullin, Leatherman, et al., 2005; Petecchia et al., 2012). In this current study, ERK inhibition by U0126 improved barrier function outright, as shown by enhancing electrical resistance and increasing dome formation in control 16HBE cell layers, a morphological phenomenon indicative of improved TJ complexes (Figure 8a–c). This ratifies the conclusion by others that MAPK signaling can play a regulatory role in 16HBE barrier function (Callaghan et al., 2020; Durgan et al., 2015; Li et al., 2015). Our results however show no effect of calcitriol on pERK (or total ERK) levels in 16HBE cell layers (Figure 9).

In the presence of TNF‐α, calcitriol and U0126 exhibited small but significant additive effects on TER (Figure 10), suggesting that calcitriol in part inhibits TNF‐α compromise of 16HBE barrier function by a pathway other than ERK. The lack of effect of calcitriol on the elevated level of pERK caused by TNF‐α (Figure 11) is further evidence of this lack of involvement of the ERK pathway in calcitriol's effect on TNF‐α ‐compromised barrier function in 16HBE cell layers. At present we have however not identified this pathway by which calcitriol is countering TNF‐α ‐induced barrier compromise. Potential candidates for this non‐ERK pathway by which calcitriol could regulate barrier function could include pathways leading to cell death, as there is extensive published literature describing calcitriol‐mediated inhibition of both apoptosis and autophagy (Langberg et al., 2009; Lyu et al., 2020; Xiong et al., 2021). However, the lack of any significant effect of TNF‐α on caspase‐3 levels in 16HBE, and the lack of effect of calcitriol on the increase in LC3B II levels caused by TNF‐α (Figure 12), suggest that this unknown calcitriol pathway is not a death pathway. Future work will focus on identifying the nature of this non‐ERK pathway by which calcitriol protects the 16HBE cell layer from TNF‐α's barrier compromising effects.

Certain studies have shown synergistic effects of Vitamins A and D on various cell properties (Anand & Kaul, 2003; Cantorna et al., 2019; Surman et al., 2016). However, to our knowledge there are no existing reports of this combination having an additive effect on epithelial barrier function. Given that these agents each produce unique effects transduced by different signaling pathways, the combination could theoretically result in an additive effect. However, in this study, using maximally effective concentrations of each vitamin, simultaneously administered calcitriol did not significantly improve on the effect of retinoic acid on transepithelial electrical resistance (Figure 13).

Further experiments are needed to more fully evaluate the mechanism involved in calcitriol's improvement of barrier function of 16HBE cell layers. However, the enhancement of control cell layer barrier function and significant abrogation of TNF‐ α's deleterious effects demonstrated by calcitriol in this study suggests that Vitamin D supplementation could play a beneficial role in protecting airway epithelial barrier function in specific disease morbidities. This is of particular importance to consider given the current COVID‐19 epidemic and disease outcomes associated with Vitamin D status. Low Vitamin D levels have been associated with an increased risk of COVID infection and hospitalization (Liu et al., 2021; Merzon et al., 2020; Munshi et al., 2021). Patients with Vitamin D deficiency were 5‐fold more likely to be infected with COVID‐19, after adjusting for age (Katz et al., 2021). Additionally, (Daneshkhah et al., 2020) Daneshkhah et al. (2020) report a potential role of Vitamin D in reducing the severity of the “cytokine storm” generated in COVID patients in terms of a Vitamin‐D‐associated reduction in pro‐inflammatory cytokine levels and C‐Reactive Protein levels. A clinical case study of four Vitamin D‐deficient COVID‐19 patients found that high dose ergocalciferol supplementation decreased hospital stay (Ohaegbulam et al., 2020). Additionally, a pilot study of COVID‐19 inpatients demonstrated that calcifediol supplementation reduced the need for ICU treatment (Entrenas Castillo et al., 2020). The ability of calcitriol to improve and protect airway epithelial barrier function in our current study suggests that Vitamin D's beneficial effects in COVID may trace not simply to effects on the virus itself but in addition to support of underlying epithelial tissue physiology before and during an infection. While it is true that research findings coming from human epithelial cell culture models cannot generate clinically applicable *conclusions* due to the limitations of the models, the studies can however validly generate very strong *hypotheses* to test further using animal models and patient‐based studies.

There is thus abundant and growing evidence to suggest a potential adjuvant clinical utility for Vitamin D supplementation in improving barrier function and reducing inflammatory response‐based damage to the airway barrier, warranting future research into the value of its use in treatment of respiratory infections such as SARS‐CoV‐2 and airway disease generally. Vitamin D therapy may reduce morbidity and thereby be pivotal in allowing a patient's own immune defenses and physiology to achieve more favorable clinical outcomes. This general issue of prophylactic and therapeutic utility of Vitamin D as well as a wider range of micronutrients in a spectrum of diseases is the subject of a very recent review on this topic (DiGuilio et al., 2022).

In summary, our results have demonstrated that: (1) calcitriol can improve normal 16HBE epithelial barrier function as well as partially protect the 16HBE cell layer from TNF‐α‐induced barrier compromise; (2) the calcitriol effects appear mediated at least in part by induced changes in the TJ complex, evidenced by effects on claudin‐2 abundance; (3) although the ERK pathway appears involved in normal 16HBE barrier function and in the compromise of that barrier function by TNF‐α, the protection accorded by calcitriol appears to proceed through a non‐ERK pathway; (4) the nature of that non‐ERK pathway in 16HBE cell layers is as yet unknown but does not appear to be a cell death‐mediating pathway.

## AUTHOR CONTRIBUTIONS

Elizabeth Rybakovsky performed the majority of cell culture, electrophysiological, radiotracer flux and Western immunoblot experiments and assisted in data analysis manuscript preparation. Katherine M. DiGuilio assisted in cell culture, electrophysiological and radiotracer flux experiments, and in data analysis and manuscript preparation and revision. Mary Carmen Valenzano performed Western immunoblot studies of particulate cell fractions. Sophie Geagen and Kaithlyn Pham assisted in electrophysiology and radiotracer flux studies and data analyses. Ronald N. Harty and James M. Mullin were responsible for study planning, experimental design, and overall manuscript preparation. J.M. Mullin assisted in cell culture and data analyses.

## FUNDING INFORMATION

Financial support for this research came in part from a research grant from the Sharpe‐Strumia Research Foundation (JMM) and NIH grant AI139392 (RNH).

## ETHICAL STATEMENT

This Study used neither animal nor human subjects. All science was conducted in an ethical manner.

## References

[phy215592-bib-0001] Aggarwal, S. , Suzuki, T. , Taylor, W. L. , Bhargava, A. , & Rao, R. K. (2011). Contrasting effects of ERK on tight junction integrity in differentiated and under‐differentiated Caco‐2 cell monolayers. The Biochemical Journal, 433(1), 51–63. 10.1042/BJ20100249 20961289 PMC4438673

[phy215592-bib-0002] Alenmyr, L. , Uller, L. , Greiff, L. , Högestätt, E. D. , & Zygmunt, P. M. (2014). TRPV4‐mediated calcium influx and ciliary activity in human native airway epithelial cells. Basic & Clinical Pharmacology & Toxicology, 114(2), 210–216. 10.1111/bcpt.12135 24034343

[phy215592-bib-0003] Amoozadeh, Y. , Dan, Q. , Xiao, J. , Waheed, F. , & Szászi, K. (2015). Tumor necrosis factor‐α induces a biphasic change in claudin‐2 expression in tubular epithelial cells: Role in barrier functions. American Journal of Physiology. Cell Physiology, 309(1), C38–C50. 10.1152/ajpcell.00388.2014 25948735 PMC4490324

[phy215592-bib-0004] Anand, P. , & Kaul, D. (2003). Vitamin D3‐dependent pathway regulates TACO gene transcription. Biochemical and Biophysical Research Communications, 310(3), 876–877. 10.1016/j.bbrc.2003.09.087 14550285

[phy215592-bib-0005] Barbin, G. , Roisin, M. P. , & Zalc, B. (2001). Tumor necrosis factor alpha activates the phosphorylation of ERK, SAPK/JNK, and P38 kinase in primary cultures of neurons. Neurochemical Research, 26(2), 107–112. 10.1023/a:1011086426652 11478736

[phy215592-bib-0006] Brenner, H. , & Schöttker, B. (2020). Vitamin D insufficiency may account for almost nine of ten COVID‐19 deaths: Time to act. Comment on: "vitamin D deficiency and outcome of COVID‐19 patients". *Nutrients* 2020, *12*, 2757. Nutrients, 12(12), 3642. 10.3390/nu12123642 33260798 PMC7761047

[phy215592-bib-0007] Bücker, R. , Zakrzewski, S. S. , Wiegand, S. , Pieper, R. , Fromm, A. , Fromm, M. , Günzel, D. , & Schulzke, J. D. (2020). Zinc prevents intestinal epithelial barrier dysfunction induced by alpha‐hemolysin‐producing Escherichia coli 536 infection in porcine colon. Veterinary Microbiology, 243, 108632. 10.1016/j.vetmic.2020.108632 32273011

[phy215592-bib-0008] Callaghan, P. J. , Rybakovsky, E. , Ferrick, B. , Thomas, S. , & Mullin, J. M. (2020). Retinoic acid improves baseline barrier function and attenuates TNF‐α‐induced barrier leak in human bronchial epithelial cell culture model, 16HBE 14o. PLoS One, 15(12), e0242536. 10.1371/journal.pone.0242536 33301441 PMC7728186

[phy215592-bib-0009] Cantorna, M. , Snyder, L. , & Arora, J. (2019). Vitamin a and vitamin D regulate the microbial complexity, barrier function and the mucosal immune responses to insure intestinal homeostasis. Critical Reviews in Biochemistry and Molecular Biology, 54(2), 184–192. 31084433 10.1080/10409238.2019.1611734 PMC6629036

[phy215592-bib-0010] Chen, H. , Lu, R. , Zhang, Y. G. , & Sun, J. (2018). Vitamin D receptor deletion leads to the destruction of tight and Adherens junctions in lungs. Tissue Barriers, 6(4), 1–13. 10.1080/21688370.2018.1540904 PMC6389123 30409076

[phy215592-bib-0011] Chowdhury, F. , Howat, W. J. , Phillips, G. J. , & Lackie, P. M. (2010). Interactions between endothelial cells and epithelial cells in a combined cell model of airway mucosa: Effects on tight junction permeability. Experimental Lung Research, 36(1), 1–11. 10.3109/01902140903026582 20128677

[phy215592-bib-0012] Colpitts, C. C. , & Baumert, T. F. (2017). Claudins in viral infection: From entry to spread. Pflügers Archiv, 469(1), 27–34. 10.1007/s00424-016-1908-4 27885488 PMC5299967

[phy215592-bib-0013] Cozens, A. L. , Yezzi, M. J. , Kunzelmann, K. , Ohrui, T. , Chin, L. , Eng, K. , Finkbeiner, W. E. , Widdicombe, J. H. , & Gruenert, D. C. (1994). CFTR expression and chloride secretion in polarized immortal human bronchial epithelial cells. American Journal of Respiratory Cell and Molecular Biology, 10(1), 38–47. 10.1165/ajrcmb.10.1.7507342 7507342

[phy215592-bib-0014] Daneshkhah, A. , Agrawal, V. , Eshein, A. , Subramanian, H. , Roy, H. K. , & Backman, V. (2020). Evidence for possible association of vitamin D status with cytokine storm and unregulated inflammation in COVID‐19 patients. Aging Clinical and Experimental Research, 32(10), 2141–2158. 10.1007/s40520-020-01677-y 32876941 PMC7465887

[phy215592-bib-0015] DiGuilio, K. M. , Rybakovsky, E. , Abdavies, R. , Chamoun, R. , Flounders, C. A. , Shepley‐McTaggart, A. , Harty, R. N. , & Mullin, J. M. (2022). Micronutrient improvement of epithelial barrier function in various disease states: A case for adjuvant therapy. International Journal of Molecular Sciences, 23(6), 2995. 10.3390/ijms23062995 35328419 PMC8951934

[phy215592-bib-0016] Droessler, L. , Cornelius, V. , Boehm, E. , Stein, L. , Brunner, N. , & Amasheh, S. (2022). Barrier perturbation in porcine Peyer's patches by tumor necrosis factor is associated with a dysregulation of Claudins. Frontiers in Physiology, 30(13), 889552. 10.3389/fphys.2022.889552 PMC9189282 35707009

[phy215592-bib-0017] Durgan, J. , Tao, G. , Walters, M. S. , Florey, O. , Schmidt, A. , Arbelaez, V. , Rosen, N. , Crystal, R. G. , & Hall, A. (2015). SOS1 and Ras regulate epithelial tight junction formation in the human airway through EMP1. EMBO Reports, 16(1), 87–96. 10.15252/embr.201439218 25394671 PMC4304732

[phy215592-bib-0018] Entrenas Castillo, M. , Entrenas Costa, L. M. , Vaquero Barrios, J. M. , Alcalá Díaz, J. F. , López Miranda, J. , Bouillon, R. , & Quesada Gomez, J. M. (2020). Effect of calcifediol treatment and best available therapy versus best available therapy on intensive care unit admission and mortality among patients hospitalized for COVID‐19: A pilot randomized clinical study. The Journal of Steroid Biochemistry and Molecular Biology, 203, 105751. 10.1016/j.jsbmb.2020.105751 32871238 PMC7456194

[phy215592-bib-0019] Fernandez‐Robredo, P. , González‐Zamora, J. , Recalde, S. , Bilbao‐Malavé, V. , Bezunartea, J. , Hernandez, M. , & Garcia‐Layana, A. (2020). Vitamin D protects against oxidative stress and inflammation in human retinal cells. Antioxidants (Basel), 9(9), 838. 10.3390/antiox9090838 32911690 PMC7555517

[phy215592-bib-0020] Furuse, M. , Sasaki, H. , & Tsukita, S. (1999). Manner of interaction of heterogeneous claudin species within and between tight junction strands. The Journal of Cell Biology, 147(4), 891–903. 10.1083/jcb.147.4.891 10562289 PMC2156154

[phy215592-bib-0021] Gitter, A. H. , Bendfeldt, K. , Schulzke, J. D. , & Fromm, M. (2000). Leaks in the epithelial barrier caused by spontaneous and TNF‐alpha‐induced single‐cell apoptosis. The FASEB Journal, 14(12), 1749–1753. 10.1096/fj.99-0898com 10973924

[phy215592-bib-0022] Groeger, S. E. , & Meyle, J. (2015). Epithelial barrier and oral bacterial infection. Periodontology 2000, 69(1), 46–67. 10.1111/prd.12094 26252401

[phy215592-bib-0023] Guttman, J. A. , & Finlay, B. B. (2009). Tight junctions as targets of infectious agents. Biochimica et Biophysica Acta, 1788(4), 832–841. 10.1016/j.bbamem.2008.10.028 19059200

[phy215592-bib-0024] Haws, C. , Krouse, M. E. , Xia, Y. , Gruenert, D. C. , & Wine, J. J. (1992). CFTR channels in immortalized human airway cells. The American Journal of Physiology, 263(6 Pt 1), L692–L707. 10.1152/ajplung.1992.263.6.L692 1282304

[phy215592-bib-0025] Hayashi, H. , Okamatsu, M. , Ogasawara, H. , Tsugawa, N. , Isoda, N. , Matsuno, K. , & Sakoda, Y. (2020). Oral supplementation of the vitamin D metabolite 25(OH)D_3_ against influenza virus infection in mice. Nutrients, 12(7), 2000. 32635656 10.3390/nu12072000 PMC7400405

[phy215592-bib-0026] Infante, M. , Buoso, A. , Pieri, M. , Lupisella, S. , Nuccetelli, M. , Bernardini, S. , Fabbri, A. , Iannetta, M. , Andreoni, M. , Colizzi, V. , & Morello, M. (2021). Low vitamin D status at admission as a risk factor for poor survival in hospitalized patients with COVID‐10: An Italian retrospective study. Journal of the American College of Nutrition, 18, 1–16. 10.1080/07315724.2021.1877580 PMC7899172 33600292

[phy215592-bib-0027] Inoue, H. , Akimoto, K. , Homma, T. , Tanaka, A. , & Sagara, H. (2020). Airway epithelial dysfunction in asthma: Relevant to epidermal growth factor receptors and airway epithelial cells. Journal of Clinical Medicine, 9(11), 3698. 10.3390/jcm9113698 33217964 PMC7698733

[phy215592-bib-0028] Katz, J. , Yue, S. , & Xue, W. (2021). Increased risk for COVID‐19 in patients with vitamin D deficiency. Nutrition, 84, 111106. 10.1016/j.nut.2020.111106 33418230 PMC7716744

[phy215592-bib-0029] Khare, D. , Godbole, N. M. , Pawar, S. D. , Mohan, V. , Pandey, G. , Gupta, S. , Kumar, D. , Dhole, T. N. , & Godbole, M. M. (2013). Calcitriol [1, 25[OH]2 D3] pre‐ and post‐treatment suppresses inflammatory response to influenza a (H1N1) infection in human lung A549 epithelial cells. European Journal of Nutrition, 52(4), 1405–1415. 10.1007/s00394-012-0449-7 23015061

[phy215592-bib-0030] Krug, S. M. , Schulzke, J. D. , & Fromm, M. (2014). Tight junction, selective permeability, and related diseases. Seminars in Cell & Developmental Biology, 36, 166–176. 10.1016/j.semcdb.2014.09.002 25220018

[phy215592-bib-0031] Langberg, M. , Rotem, C. , Fenig, E. , Koren, R. , & Ravid, A. (2009). Vitamin D protects keratinocytes from deleterious effects of ionizing radiation. The British Journal of Dermatology, 160(1), 151–161. 10.1111/j.1365-2133.2008.08797.x 18717671

[phy215592-bib-0032] Lee, C. , Lau, E. , Chusilp, S. , Filler, R. , Li, B. , Zhu, H. , Yamoto, M. , & Pierro, A. (2019). Protective effects of vitamin D against injury in intestinal epithelium. Pediatric Surgery International, 35(12), 1395–1401. 10.1007/s00383-019-04586-y 31612340

[phy215592-bib-0033] Li, W. , Dong, H. , Zhao, H. , Song, J. , Tang, H. , Yao, L. , Liu, L. , Tong, W. , Zou, M. , Zou, F. , & Cai, S. (2015). 1,25‐ Dihydroxyvitamin D3 prevents toluene diisocyanate‐induced airway epithelial barrier disruption. International Journal of Molecular Medicine, 36(1), 263–270. 10.3892/ijmm.2015.2214 25998793

[phy215592-bib-0034] Liu, N. , Sun, J. , Wang, X. , Zhang, T. , Zhao, M. , & Li, H. (2021). Low vitamin D status is associated with coronavirus disease 2019 outcomes: A systematic review and meta‐analysis. International Journal of Infectious Diseases, 104, 58–64. 10.1016/j.ijid.2020.12.077 33401034 PMC7833186

[phy215592-bib-0035] Lyu, N. , Zhang, J. , Dai, Y. , Xiang, J. , Li, Y. , & Xu, J. (2020). Calcitriol inhibits apoptosis via activation of autophagy in hyperosmotic stress stimulated corneal epithelial cells in vivo and in vitro. Experimental Eye Research, 200, 108210. 10.1016/j.exer.2020.108210 32896533

[phy215592-bib-0036] Ma, S. W. , Ende, J. A. , Alvarado, R. , Christensen, J. M. , Kalish, L. , Sacks, R. , Campbell, R. , Rimmer, J. , & Harvey, R. (2020). Topical vitamin D may modulate human Sinonasal mucosal responses to house dust mite antigen. American Journal of Rhinology & Allergy, 34(4), 471–481. 10.1177/1945892420905432 32046501

[phy215592-bib-0037] Meckel, K. , Li, Y. C. , Lim, J. , Kocherginsky, M. , Weber, C. , Almoghrabi, A. , Chen, X. , Kaboff, A. , Sadiq, F. , Hanauer, S. B. , Cohen, R. D. , Kwon, J. , Rubin, D. T. , Hanan, I. , Sakuraba, A. , Yen, E. , Bissonnette, M. , & Pekow, J. (2016). Serum 25‐hydroxyvitamin D concentration is inversely associated with mucosal inflammation in patients with ulcerative colitis. The American Journal of Clinical Nutrition, 104(1), 113–120. 10.3945/ajcn.115.123786 27281309 PMC4919525

[phy215592-bib-0038] Merzon, E. , Tworowski, D. , Gorohovski, A. , Vinker, S. , Golan Cohen, A. , Green, I. , & Frenkel‐Morgenstern, M. (2020). Low plasma 25(OH) vitamin D level is associated with increased risk of COVID‐19 infection: An Israeli population‐based study. The FEBS Journal, 287(17), 3693–3702. 10.1111/febs.15495 32700398 PMC7404739

[phy215592-bib-0039] Mohanty, S. , Kamolvit, W. , Hertting, O. , & Brauner, A. (2020). Vitamin D strengthens the bladder epithelial barrier by inducing tight junction proteins during E. coli urinary tract infection. Cell and Tissue Research, 380(3), 669–673. 10.1007/s00441-019-03162-z 31930458 PMC7242269

[phy215592-bib-0040] Mullin, J. M. , Agostino, N. , Rendon‐Huerta, E. , & Thornton, J. J. (2005). Keynote review: Epithelial and endothelial barriers in human disease. Drug Discovery Today, 10(6), 395–408. 10.1016/S1359-6446(05)03379-9 15808819

[phy215592-bib-0041] Mullin, J. M. , Leatherman, J. M. , Valenzano, M. C. , Huerta, E. R. , Verrechio, J. , Smith, D. M. , Snetselaar, K. , Liu, M. , Francis, M. K. , & Sell, C. (2005). Ras mutation impairs epithelial barrier function to a wide range of nonelectrolytes. Molecular Biology of the Cell, 16(12), 5538–5550. 10.1091/mbc.e05-04-0294 16176977 PMC1289400

[phy215592-bib-0042] Munshi, R. , Hussein, M. H. , Toraih, E. A. , Elshazli, R. M. , Jardak, C. , Sultana, N. , Youssef, M. R. , Omar, M. , Attia, A. S. , Fawzy, M. S. , Killackey, M. , Kandil, E. , & Duchesne, J. (2021). Vitamin D insufficiency as a potential culprit in critical COVID‐19 patients. Journal of Medical Virology, 93(2), 733–740. 10.1002/jmv.26360 32716073

[phy215592-bib-0043] Murdaca, G. , Pioggia, G. , & Negrini, S. (2020). Vitamin D and Covid‐19: An update on evidence and potential therapeutic implications. Clinical and Molecular Allergy, 18(1), 23. 10.1186/s12948-020-00139-0 33292313 PMC7675394

[phy215592-bib-0044] Nelson, C. D. , Powell, J. L. , Price, D. M. , Hersom, M. J. , Yelich, J. V. , Drewnoski, M. E. , Bird, S. L. , & Bridges, G. A. (2016). Assessment of serum 25‐hydroxyvitamin D concentrations of beef cows and calves across seasons and geographical locations. Journal of Animal Science, 94(9), 3958–3965. 10.2527/jas.2016-0611 27898926

[phy215592-bib-0045] Ohaegbulam, K. C. , Swalih, M. , Patel, P. , Smith, M. A. , & Perrin, R. (2020). Vitamin D supplementation in COVID‐19 patients: A clinical case series. American Journal of Therapeutics, 27(5), e485–e490. 10.1097/MJT.0000000000001222 32804682 PMC7473790

[phy215592-bib-0046] Pan, W. , Borovac, J. , Spicer, Z. , Hoenderop, J. G. , Bindels, R. J. , Shull, G. E. , Doschak, M. R. , Cordat, E. , & Alexander, R. T. (2012). The epithelial sodium/proton exchanger, NHE3, is necessary for renal and intestinal calcium (re)absorption. American Journal of Physiology. Renal Physiology, 302(8), F943–F956. 21937605 10.1152/ajprenal.00504.2010 PMC3330715

[phy215592-bib-0047] Peralta Soler, A. , Mullin, J. M. , Knudsen, K. A. , & Marano, C. W. (1996). Tissue remodeling during tumor necrosis factor‐induced apoptosis in LLC‐PK1 renal epithelial cells. The American Journal of Physiology, 270(5 Pt2), F869–F879. 10.1152/ajprenal.1996.270.5.F869 8928850

[phy215592-bib-0048] Petecchia, L. , Sabatini, F. , Usai, C. , Caci, E. , Varesio, L. , & Rossi, G. A. (2012). Cytokines induce tight junction disassembly in airway cells via an EGFR‐dependent MAPK/ERK1/2‐pathway. Laboratory Investigation, 92(8), 1140–1148. 10.1038/labinvest.2012.67 22584669

[phy215592-bib-0049] Randolph, R. K. , & Simon, M. (1993). Characterization of retinol metabolism in cultured human epidermal keratinocytes. The Journal of Biological Chemistry, 268(13), 9198–9205. 8486621

[phy215592-bib-0050] Rosenthal, R. , Günzel, D. , Theune, D. , Czichos, C. , Schulzke, J. D. , & Fromm, M. (2017). Water channels and barriers formed by claudins. Annals of the New York Academy of Sciences, 1397(1), 100–109. 10.1111/nyas.13383 28636801

[phy215592-bib-0051] Sabetta, J. , DePetrillo, P. , Cipriani, R. , Smardin, J. , Burns, L. , & Landry, M. (2010). Serum 25‐hydroxyvitamin d and the incidence of acute viral respiratory tract infections in healthy adults. PLoS One, 5(6), e11088. 10.1371/journal.pone.0011088 20559424 PMC2885414

[phy215592-bib-0052] Sawada, N. (2013). Tight junction‐related human diseases. Pathology International, 63(1), 1–12. 10.1111/pin.12021 23356220 PMC7168075

[phy215592-bib-0053] Schlingmann, B. , Overgaard, C. E. , Molina, S. A. , Lynn, K. S. , Mitchell, L. A. , Dorsainvil White, S. , Mattheyses, A. L. , Guidot, D. M. , Capaldo, C. T. , & Koval, M. (2016). Regulation of claudin/zonula occludens‐1 complexes by hetero‐claudin interactions. Nature Communications, 25(7), 12276. 10.1038/ncomms12276 PMC4962485 27452368

[phy215592-bib-0054] Sekiyama, A. , Gon, Y. , Terakado, M. , Takeshita, I. , Kozu, Y. , Maruoka, S. , Matsumoto, K. , & Hashimoto, S. (2012). Glucocorticoids enhance airway epithelial barrier integrity. International Immunopharmacology, 12(2), 350–357. 10.1016/j.intimp.2011.12.006 22210372

[phy215592-bib-0055] Shashikanth, N. , France, M. M. , Xiao, R. , Haest, X. , Rizzo, H. E. , Yeste, J. , Reiner, J. , & Turner, J. R. (2022). Tight junction channel regulation by interclaudin interference. Nature Communications, 13(1), 3780. 10.1038/s41467-022-31587-8 PMC9246906 35773259

[phy215592-bib-0056] Shintani, Y. , Maruoka, S. , Gon, Y. , Koyama, D. , Yoshida, A. , Kozu, Y. , Kuroda, K. , Takeshita, I. , Tsuboi, E. , Soda, K. , & Hashimoto, S. (2015). Nuclear factor erythroid 2‐related factor 2 (Nrf2) regulates airway epithelial barrier integrity. Allergology International, 64(Suppl), S54–S63. 10.1016/j.alit.2015.06.004 26344081

[phy215592-bib-0057] Sonoda, N. , Furuse, M. , Sasaki, H. , Yonemura, S. , Katahira, J. , Horiguchi, Y. , & Tsukita, S. (1999). Clostridium perfringens enterotoxin fragment removes specific claudins from tight junction strands: Evidence for direct involvement of claudins in tight junction barrier. The Journal of Cell Biology, 147(1), 195–204. 10.1083/jcb.147.1.195 10508866 PMC2164970

[phy215592-bib-0058] Surman, S. L. , Penkert, R. R. , Jones, B. G. , Sealy, R. E. , & Hurwitz, J. L. (2016). Vitamin supplementation at the time of immunization with a cold‐adapted influenza virus vaccine corrects poor mucosal antibody responses in mice deficient for vitamins a and D. Clinical and Vaccine Immunology, 23(3), 219–227. 10.1128/CVI.00739-15 26740391 PMC4783424

[phy215592-bib-0059] Sweerus, K. , Lachowicz‐Scroggins, M. , Gordon, E. , LaFemina, M. , Huang, X. , Parikh, M. , Kanegai, C. , Fahy, J. V. , & Frank, J. A. (2017). Claudin‐18 deficiency is associated with airway epithelial barrier dysfunction and asthma. The Journal of Allergy and Clinical Immunology, 139(1), 72–81.e1. 10.1016/j.jaci.2016.02.035 27215490 PMC5073041

[phy215592-bib-0060] Torres‐Flores, J. M. , & Arias, C. F. (2015). Tight junctions go viral! Viruses, 7(9), 5145–5154. 10.3390/v7092865 26404354 PMC4584309

[phy215592-bib-0061] Uddin, M. , Seumois, G. , Lau, L. C. , Rytila, P. , Davies, D. E. , & Djukanovic, R. (2008). Enhancement of neutrophil function by the bronchial epithelium stimulated by epidermal growth factor. The European Respiratory Journal, 31(4), 714–724. 10.1183/09031936.00144307 18094008

[phy215592-bib-0062] Valenzano, M. C. , DiGuilio, K. , Mercado, J. , Teter, M. , To J , Ferraro, B. , Mixson, B. , Manleu, I. , Baker, V. , Moore, B. A. , Wertheimer, J. , & Mullin, J. M. M. (2015). Remodeling of tight junctions and enhancement of barrier integrity of the CACO‐2 intestinal epithelial cell layer by micronutrients. PLoS One, 10(7), e0133926. 26226276 10.1371/journal.pone.0133926 PMC4520484

[phy215592-bib-0063] Vargas‐Robles, H. , Castro‐Ochoa, K. F. , Citalán‐Madrid, A. F. , & Schnoor, M. (2019). Beneficial effects of nutritional supplements on intestinal epithelial barrier functions in experimental colitis models. World Journal of Gastroenterology, 25(30), 4181–4198. 10.3748/wjg.v25.i30.4181 31435172 PMC6700707

[phy215592-bib-0064] Wine, J. J. , Finkbeiner, W. E. , Haws, C. , Krouse, M. E. , Moon, S. , Widdicombe, J. H. , & Xia, Y. (1994). CFTR and other Cl‐channels in human airway cells. The Japanese Journal of Physiology, 44(Suppl 2), S199–S205. 7752526

[phy215592-bib-0065] Xatzipsalti, M. , & Papadopoulos, N. G. (2007). Cellular and animals models for rhinovirus infection in asthma. Contributions to Microbiology, 14, 33–41. 10.1159/000107053 17684331

[phy215592-bib-0066] Xiong, Y. , Zhou, F. , Liu, Y. , Yi, Z. , Wang, X. , Wu, Y. , & Gong, P. (2021). 1α,25‐Dihydroxyvitamin D3 promotes angiogenesis by alleviating AGEs‐induced autophagy. Archives of Biochemistry and Biophysics, 712, 109041. 10.1016/j.abb.2021.109041 34560065

[phy215592-bib-0067] Yamada, S. , & Kanda, Y. (2019). Retinoic acid promotes barrier functions in human iPSC‐derived intestinal epithelial monolayers. Journal of Pharmacological Sciences, 140(4), 337–344. 10.1016/j.jphs.2019.06.012 31399314

[phy215592-bib-0068] Zhu, J. , Rogers, A. V. , Burke‐Gaffney, A. , Hellewell, P. G. , & Jeffery, P. K. (1999). Cytokine‐induced airway epithelial ICAM‐1 upregulation: Quantification by high‐resolution scanning and transmission electron microscopy. The European Respiratory Journal, 13(6), 1318–1328. 10.1183/09031936.99.13613299 10445607

